# Exploring the relationship between anthropomorphism and theory‐of‐mind in brain and behaviour

**DOI:** 10.1002/hbm.25542

**Published:** 2021-07-01

**Authors:** Ruud Hortensius, Michaela Kent, Kohinoor M. Darda, Laura Jastrzab, Kami Koldewyn, Richard Ramsey, Emily S. Cross

**Affiliations:** ^1^ Department of Psychology Utrecht University Utrecht The Netherlands; ^2^ Institute of Neuroscience and Psychology, School of Psychology University of Glasgow Glasgow Scotland UK; ^3^ Faculty of Neuroscience, Brain and Mind Institute University of Western Ontario London Ontario Canada; ^4^ Department of Cognitive Science Macquarie University Sydney New South Wales Australia; ^5^ Wales Institute for Cognitive Neuroscience, School of Psychology Bangor University Bangor Wales UK; ^6^ Department of Psychology Macquarie University Sydney New South Wales Australia

**Keywords:** anthropomorphism, mind perception, social cognition, theory‐of‐mind

## Abstract

The process of understanding the minds of other people, such as their emotions and intentions, is mimicked when individuals try to understand an artificial mind. The assumption is that anthropomorphism, attributing human‐like characteristics to non‐human agents and objects, is an analogue to theory‐of‐mind, the ability to infer mental states of other people. Here, we test to what extent these two constructs formally overlap. Specifically, using a multi‐method approach, we test *if* and *how* anthropomorphism is related to theory‐of‐mind using brain (Experiment 1) and behavioural (Experiment 2) measures. In a first exploratory experiment, we examine the relationship between dispositional anthropomorphism and activity within the theory‐of‐mind brain network (*n* = 108). Results from a Bayesian regression analysis showed no consistent relationship between dispositional anthropomorphism and activity in regions of the theory‐of‐mind network. In a follow‐up, pre‐registered experiment, we explored the relationship between theory‐of‐mind and situational and dispositional anthropomorphism in more depth. Participants (*n* = 311) watched a short movie while simultaneously completing situational anthropomorphism and theory‐of‐mind ratings, as well as measures of dispositional anthropomorphism and general theory‐of‐mind. Only situational anthropomorphism predicted the ability to understand and predict the behaviour of the film's characters. No relationship between situational or dispositional anthropomorphism and general theory‐of‐mind was observed. Together, these results suggest that while the constructs of anthropomorphism and theory‐of‐mind might overlap in certain situations, they remain separate and possibly unrelated at the personality level. These findings point to a possible dissociation between brain and behavioural measures when considering the relationship between theory‐of‐mind and anthropomorphism.

## Graphical abstract

Is trying to understand an artificial mind the same as trying to understand a human mind? Using a multi‐method approach, we test *if* and *how* the process of anthropomorphism overlaps with that of theory‐of‐mind using brain and behavioural measures. While dispositional anthropomorphism did not capture variation in theory‐of‐mind network activity, situational anthropomorphism did influence how people understand and predict the ongoing actions of an agent.

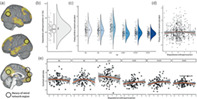

## 1 INTRODUCTION

Our abilities to infer and predict observed and hidden mental states of other people, such as their intentions, beliefs, and emotions, shape our ongoing social interactions. This process, which has been termed theory‐of‐mind (Premack & Woodruff, 1978) or mentalising (Frith, Wolpert, Frith, & Frith, 2003), is fundamental to human social life. Similar attributions of mental states are also made towards non‐human agents and objects, and this process, termed anthropomorphism, actively contributes to engagement with these agents and objects (Broadbent, 2017; Hortensius & Cross, 2018). Anthropomorphism has been described as the extension of theory‐of‐mind to non‐human agents (Atherton & Cross, 2018). To successfully humanise behaviours of non‐human agents, or to anthropomorphise, has been suggested to rely on similar steps to those used when understanding the agent's intentions and, therefore, may be closely connected to theory‐of‐mind (Epley, Waytz, & Cacioppo, 2007). Anthropomorphism may be a foundation from which to improve aberrant theory‐of‐mind (Atherton & Cross, 2018). While at first inspection, these processes appear to overlap at a basic construct level, whether or not anthropomorphism is a partial or complete analogue of theory‐of‐mind remains unknown. The perception of another's mind, whether human or artificial in nature, has important consequences for social behaviour (Waytz, Gray, Epley, & Wegner, 2010). For instance, mind perception is required for judgements of agency over an agent's actions in the case of moral transgressions (Bigman, Waytz, Alterovitz, & Gray, 2019; K. Gray, Young, & Waytz, 2012). As mind perception and its consequences differ per individual (Waytz, Cacioppo, & Epley, 2010a; Waytz, Gray, et al., 2010), distinguishing these processes at behavioural and brain levels is vital in order to understand their functional meaning and consequences.

Anthropomorphism extends beyond just seeing human features (e.g., eyes or hands) in non‐human agents and objects. It also involves actively attributing human mental states (e.g., emotions, intentionality) to non‐human agents and objects (Epley et al., 2007). The overlap with theory‐of‐mind is already evident in this definition. Anthropomorphism is not only driven by perceptual factors (e.g., stimulus cues such as the presence of a face), but also by a motivation in the observer to understand and predict the environment (Waytz, Cacioppo, & Epley, ). This effectance motivation, as coined by White (1959), is related to exploration and mastery of the environment, and describes the drive to make sense of a world with uncertain perception‐action links. For example, effectance motivation influences one's interest and explanations when observing the actions of an unfamiliar robot. In a series of experiments, Waytz, Morewedge, and colleagues (2010) showed that this need to understand and predict the world not only increases anthropomorphism, but that anthropomorphism also fulfils this need. Effectance motivation is critical to the perception of other minds, as this motivation not only drives interactions with non‐human agents and objects, but also interactions with other humans (Waytz, Gray, et al., 2010). Indeed, prediction of another individual's behaviour is central to theory‐of‐mind and its underlying neural network (Koster‐Hale & Saxe, 2013). As astutely described by Heider (1958), extracting invariance out of variance, inferring intentions and emotions from the behaviour of an agent, is a fundamental feature of social cognition. Both theory‐of‐mind and anthropomorphism are ways to achieve this.

Further support for a relation between anthropomorphism and theory‐of‐mind comes from neuroimaging studies. These studies show that brain regions activated when individuals engage in anthropomorphism include regions considered to be part of the theory‐of‐mind network (Hortensius & Cross, 2018). This network consists of bilateral temporoparietal junction (TPJ), the precuneus and parts of the medial prefrontal cortex, and has consistently been implicated in tasks examining how we understand and predict the mental states of other agents (Carrington & Bailey, 2009; Schurz, Radua, Aichhorn, Richlan, & Perner, 2014). For instance, parts of the TPJ are not only activated during standardised theory‐of‐mind tasks, but also when observing non‐human social animations that can trigger attributions of agency, intent, and other mental states (Schurz, Tholen, Perner, Mars, & Sallet, 2017). Initial evidence suggests a link between activity in these regions and more direct measures of anthropomorphism. Activity in the left TPJ and the Pprecuneus is higher for individuals with an increased tendency to report the movement of an animated character as biological in origin (Chaminade, Hodgins, & Kawato, 2007). Activity in the ventromedial prefrontal cortex (vMPFC) is increased for unpredictable gadgets compared to predictable gadgets, with the former also leading to more human mental state attributions (Waytz, Morewedge, et al., 2010). Exploratory results suggest that a disposition to attribute human states to non‐human animals is correlated with left TPJ grey matter (GM) volume (Cullen, Kanai, Bahrami, & Rees, 2014). While these initial studies provide first insights into the link between anthropomorphism and theory‐of‐mind network engagement, a question remains as to whether activity within and across these regions of the theory‐of‐mind network is enhanced in individuals who are more likely to attribute human mental states to non‐human agents and objects.

Both theory‐of‐mind and anthropomorphism are multi‐dimensional constructs, with both terms often used to describe a range of processes. Theory‐of‐mind is a popular topic of study in the field of social cognition and has previously been linked to a number of behaviours from moral development (Killen, Mulvey, Richardson, Jampol, & Woodward, 2011) to empathy (Baron‐Cohen & Wheelwright, 2004). While partial overlap could occur between the involved subprocesses, theory‐of‐mind remains an umbrella term that involves a host of cognitive and brain mechanisms, which may vary by task (Schaafsma, Pfaff, Spunt, & Adolphs, 2015). Different tasks measure different constructs related to theory‐of‐mind (François & Rossetti, 2020). Often used as a catch‐all term, anthropomorphism, too, is a broad concept that can vary depending on the agent, object and situation (e.g., Ruijten, Haans, Ham, & Midden, 2019). Moreover, there is large variation in the extent that people anthropomorphise (Epley et al., 2007; Waytz, Cacioppo, & Epley, 2010a). For this reason, we cast a wide net and use several proxies for both concepts with the idea that the overlap between theory‐of‐mind and anthropomorphism can be dependent on the task or situation a person is experiencing as well as their personality.

Here, we tease apart anthropomorphism and theory‐of‐mind at the behavioural and brain level in order to better understand the potential overlap between these aspects of mind perception. Across two experiments, we tested this link using a multi‐method approach thereby unpacking situational and dispositional aspects of mind perception. Instead of one‐off judgements of an agent's mind, we use behavioural and brain measures of dynamic mind perception that involve understanding, predicting, and updating of the inferred internal states of an agent over time. In a first exploratory neuroimaging experiment, we tested if an individual's tendency to engage in anthropomorphism modulates activity in the theory‐of‐mind network (*n* = 108). Specifically, we tested if activity in the theory‐of‐mind network during the observation of a short social animation is higher for individuals who are overall more disposed to attribute a mind, consciousness, free will, emotions and intentions to natural entities, non‐human animals, and technological devices. With this experiment, we asked whether higher levels of dispositional anthropomorphism are associated with higher levels of activity in the theory‐of‐mind network (i.e., a positive linear relationship). In a second, preregistered, experiment, we explored a possible relationship between theory‐of‐mind and situational and dispositional anthropomorphism in more detail, using behavioural measures in a representational UK sample (*n* = 311). We tested if an individual's tendency to anthropomorphise in general (dispositional anthropomorphism) or during the observation of a short social animation (situational anthropomorphism) is predictive of the ability to understand and predict the behaviour of the characters in this animation. Using a proxy for general theory‐of‐mind, we tested if these anthropomorphism indices are also related to the understanding of false beliefs. Previous literature has suggested that in order to better understand the concept of theory‐of‐mind, it must be broken down into a collection of more simple and specific processes (Schaafsma et al., 2015). Herein we use the term “general theory‐of‐mind” to refer and relate to the classic and very broad concept of cognitive theory‐of‐mind that can be measured by the understanding of false beliefs.

## 2 EXPERIMENT 1

### 2.1 Materials and methods

#### 2.1.1 Data statement

The data used for Experiment 1 were sampled across five separate studies undertaken by the authors at the Institute of Neuroscience and Psychology at the University of Glasgow and the School of Psychology at Bangor University, for which data acquisition was completed at the beginning of 2020. In each study, besides the main experimental task, participants completed a functional localiser that mapped the theory‐of‐mind network and a measure of dispositional anthropomorphism. For each study, we included all available data selecting all participants that completed both the functional localiser and questionnaire. As two of the studies share MRI acquisition parameters, we combined these studies into one dataset. In total, we analysed four complete datasets each with different MRI acquisition parameters. Dataset 1 contains data from Cross et al. (2019), Datasets 2 ‐ 4 contain data from completed studies or from studies with the first experiments completed.

#### 2.1.2 Participants

A total of 108 participants were included in the final analyses, with sample sizes from each study as follows: Dataset 1: *n* = 29; Dataset 2: *n* = 35; Dataset 3: *n* = 22; Dataset 4: *n* = 22. The total sample consisted of 54 women and 54 men, aged between 18 and 43 years old (Table S1). Participants were recruited primarily through the University of Glasgow and Bangor University participation pools, and by word‐of‐mouth. For all studies, participants received verbal and written information prior to the study, provided written informed consent before beginning any study, and were naive to the goal of the study. On completion of the study, experimenters debriefed the participants and answered any questions before reimbursing participants for their time (ranging from £12 to £60). Study procedures were approved by either the Centre for Cognitive Neuroimaging and Research Ethics Committee of the College of Science and Engineering at the University of Glasgow (protocol numbers: 300170226, 300180084, 300180110, 300180151, 300180208, 300180301) or the Bangor Imaging Unit and the Bangor University School of Psychology Research Ethics Committee (protocol number: 2017–16209) and carried out in accordance with the standards set by the Declaration of Helsinki.

#### 2.1.3 Dispositional anthropomorphism

To measure dispositional anthropomorphism, the Individual Differences in Anthropomorphism Questionnaire (IDAQ) was used (Waytz, Cacioppo, & Epley, 2010a). This questionnaire consists of 15 anthropomorphic items for which participants provide a rating on the extent that natural entities, non‐human animals, and technological devices have a mind of their own, consciousness, free will and intentions, and experience emotion (e.g., “to what extent does the average robot have consciousness?”). There were also 15 nonanthropomorphic items (IDAQ‐NA) for which participants provide a rating of functional features of a stimulus (good‐looking, active, useful, lethargic, durable; e.g., "to what extent is the average camera lethargic?"). These items provide a control and measure dispositional attribution in general. Responses are rated on a scale from 0 (not at all) to 10 (very much). Participants completed the IDAQ at the end of the fMRI session. The individual score of dispositional anthropomorphism was calculated following the method outlined by Waytz, Cacioppo, and Epley (2010). The reliability of IDAQ scale, Cronbach's *α* = .80, 95% confidence interval [.75–.86] (Table S2), and the IDAQ‐NA, *α* = .58 [.47–.70] were comparable with previous findings (Waytz et al., 2018; Waytz, Cacioppo, & Epley, 2010a).

#### 2.1.4 Theory‐of‐mind network localiser

We used an established localiser that reliably maps the theory‐of‐mind network (Jacoby, Bruneau, Koster‐Hale, & Saxe, 2016). Participants passively viewed a 5.6 min animated film (“Partly Cloudy,” (https://www.pixar.com/partly-cloudy#partly-cloudy-1). The film depicts how “[b]abies both human and animal are created up in the stratosphere, by the clouds themselves. One cloud specializes in “dangerous” babies, creating a challenge for his loyal stork that has to deliver them.” (IMDB, http://www.imdb.com/title/tt1425244/). The film contains scenes that trigger mentalising as well as scenes that show the main characters experiencing pain. Contrasting mentalising events with pain events identifies the theory‐of‐mind network, while the reverse contrast identifies the pain matrix, a network involved in emotional reactivity to observed pain (Jacoby et al., 2016; Richardson, Lisandrelli, Riobueno‐Naylor, & Saxe, 2018). The latter was used as a control network to test the specificity in the relationship between dispositional anthropomorphism and ToM network engagement.

#### 2.1.5 MRI data acquisition

Data were acquired with a 3‐Tesla Philips Achieva full‐body MRI scanner using a SENSE phased‐array 32‐channel head coil at Bangor University (Dataset 1) and a 3‐Tesla Siemens Tim Trio MRI scanner with a 32‐channel head coil and integrated parallel imaging techniques at the Centre for Cognitive Neuroimaging, University of Glasgow (Datasets 2–4). Participants were provided with earplugs and headphones to attenuate scanner noise and allow auditory sound during the functional localiser. Foam padding or inflatable cushions were used to reduce head movements. Each participant underwent both an anatomical and functional localiser scan while in the scanner, either in one (Datasets 2–4) or two consecutive sessions (Dataset 1).

There were slight differences between MRI parameters between datasets. Complete details can be found in Table S3. Here, we highlight the relevant parameters. Functional images were acquired using an echo planar image (EPI) sequence (Dataset 1: TR = 2,000 ms; TE = 30 ms; 32 slices per volume; 3 × 3 × 3.5 voxels; no gap; Dataset 2: TR = 2,000 ms; TE = 30 ms; 37 slices per volume; 3 mm isotropic voxels, no gap), multi‐band EPI (Dataset 3: TR = 2,000 ms; TE = 26 ms; 68 slices per volume; 2 mm isotropic voxels, no gap), and a multi‐echo EPI sequence (Dataset 4: TR = 2,000 ms; TE = 13/31 ms; 32 slices per volume; 2.75 × 2.75 × 4 mm voxels, no gap). The entire cerebral cortex was covered in all datasets. A three‐dimensional T1‐weighted (T1w) imaging sequence scan was collected (Dataset 1: 1 mm isotropic resolution, TR = 12 ms, TE = 3.47/5.15/6.83/8.52/10.20 ms, FA = 8°, field of view = 240 × 240 mm^2^; Datasets 2–4: 1 mm isotropic resolution, TR = 2,300 ms; TE = 30 ms; FA = 9°; field of view = 192 × 256 mm^2^). For Datasets 3 and 4, a field map was collected in the same session (Dataset 3: 3.28 × 3.28 × 3.3 mm voxels, TR = 488 ms, TE = 4.92/7.38 ms, FA = 60°, field of view = 192 × 192 mm^2^; Dataset 4: 2.75 × 2.75 × 4 mm voxels, TR = 488 ms, TE = 4.26/6.72, FA = 90°, field of view = 220 × 220 mm^2^).

#### 2.1.6 fMRI preprocessing

Before preprocessing, image‐quality metrics were calculated using MRIQC (version 0.14.2) (Esteban et al., 2017). Comparison of these metrics revealed similar signal and data quality across datasets (Table S4). Signal‐to‐noise ratio ranged from 3.59 to 6.77 across datasets, while mean ± *SD* framewise displacement (FD; Power et al., 2014) was 0.126 ± 0.050 (Dataset 1), 0.112 ± 0.053 (Dataset 2), 0.093 ± 0.048 (Dataset 3), and 0.163 ± 0.063 (Dataset 4).

Results included in this manuscript come from preprocessing performed using *fMRIPrep* 1.5.2 ( Esteban, Markiewicz, Blair, et al., 2019; Esteban, Markiewicz, DuPre, et al., 2019; RRID:SCR_016216), which is based on *Nipype* 1.3.1 (Gorgolewski et al. (2011); Gorgolewski et al. (2017); RRID:SCR_002502).

##### Anatomical data preprocessing

The T1w image (or images for Dataset 1) was corrected for intensity non‐uniformity (INU) with N4BiasFieldCorrection ( Tustison et al., 2010), distributed with antsApplyTransforms (ANTs) 2.2.0 (Avants, Epstein, Grossman, & Gee, 2008, RRID:SCR_004757), and for Datasets 2–4 used as T1w‐reference throughout the workflow. The T1w‐reference was then skull‐stripped with a *Nipype* implementation of the antsBrainExtraction.sh workflow (from ANTs), using OASIS30ANTs as target template. Brain tissue segmentation of cerebrospinal fluid (CSF), white‐matter (WM), and GM was performed on the brain‐extracted T1w using fast (FSL 5.0.9, RRID:SCR_002823, Zhang, Brady, & Smith, 2001). For Dataset 1, a T1w‐reference map was computed after registration of five T1w images (after INU‐correction) using mri_robust_template (FreeSurfer 6.0.1, Reuter, Rosas, & Fischl, 2010). Volume‐based spatial normalisation to one standard space (MNI152NLin2009cAsym) was performed through nonlinear registration with antsRegistration (ANTs 2.2.0), using brain‐extracted versions of both T1w reference and the T1w template. The following template was selected for spatial normalisation: *ICBM 152 Nonlinear Asymmetrical template version 2009c* (Fonov, Evans, McKinstry, Almli, and Collins (2009), RRID:SCR_008796; TemplateFlow ID: MNI152NLin2009cAsym).

##### Functional data preprocessing

Before preprocessing, the dual‐echo images of Dataset 4 were summed. For the BOLD run, the following preprocessing was performed. First, a reference volume and its skull‐stripped version were generated using a custom methodology of *fMRIPrep*. For Datasets 1 and 2, a deformation field to correct for susceptibility distortions was estimated based on *fMRIPrep*'s *fieldmap‐less* approach. The deformation field is that resulting from co‐registering the BOLD reference to the same‐subject T1w‐reference with its intensity inverted (Huntenburg, 2014; Wang et al., 2017). Registration is performed with antsRegistration (ANTs 2.2.0), and the process regularised by constraining deformation to be nonzero only along the phase‐encoding direction, and modulated with an average fieldmap template (Treiber et al., 2016). For Datasets 3 and 4, a deformation field to correct for susceptibility distortions was estimated based on a field map that was co‐registered to the BOLD reference, using a custom workflow of *fMRIPrep* derived from D. Greve's epidewarp.fsl script and further improvements of HCP pipelines (Glasser et al., 2013). Based on the estimated susceptibility distortion, an unwrapped BOLD reference was calculated for a more accurate co‐registration with the anatomical reference. The BOLD reference was then co‐registered to the T1w reference using flirt (FSL 5.0.9, Jenkinson & Smith, 2001) with the boundary‐based registration (Greve & Fischl, 2009) cost‐function. Co‐registration was configured with nine degrees of freedom to account for distortions remaining in the BOLD reference. Head‐motion parameters with respect to the BOLD reference (transformation matrices, and six corresponding rotation and translation parameters) are estimated before any spatiotemporal filtering using mcflirt (FSL 5.0.9, Jenkinson, Bannister, Brady, & Smith, 2002). For Datasets 2 and 3, BOLD runs were slice‐time corrected using 3dTshift from AFNI 20160207 (Cox & Hyde, 1997, RRID:SCR_005927). The BOLD time‐series (including slice‐timing correction when applied) were resampled onto their original, native space by applying a single, composite transform to correct for head‐motion and susceptibility distortions. These resampled BOLD time‐series will be referred to as *preprocessed BOLD in original space*, or just *preprocessed BOLD*. The BOLD time‐series were resampled into standard space, generating a *preprocessed BOLD run in [“MNI152NLin2009cAsym”] space*. First, a reference volume and its skull‐stripped version were generated using a custom methodology of *fMRIPrep*. Several confounding time‐series were calculated based on the *preprocessed BOLD*: FD, DVARS and three region‐wise global signals. FD and DVARS are calculated for each functional run, both using their implementations in *Nipype* (following the definitions by Power et al. (2014)). The three global signals are extracted within the CSF, the WM, and the whole‐brain masks. Additionally, a set of physiological regressors were extracted to allow for component‐based noise correction (*CompCo*, Behzadi, Restom, Liau, & Liu, 2007). Principal components are estimated after high‐pass filtering the *preprocessed BOLD* time‐series (using a discrete cosine filter with 128 s cut‐off) for the two *CompCor* variants: temporal (tCompCor) and anatomical (aCompCor). tCompCor components are then calculated from the top 5% variable voxels within a mask covering the subcortical regions. This subcortical mask is obtained by heavily eroding the brain mask, which ensures it does not include cortical GM regions. For aCompCor, components are calculated within the intersection of the aforementioned mask and the union of CSF and WM masks calculated in T1w space, after their projection to the native space of each functional run (using the inverse BOLD‐to‐T1w transformation). Components are also calculated separately within the WM and CSF masks. For each CompCor decomposition, the *k* components with the largest singular values are retained, such that the retained components' time series are sufficient to explain 50% of variance across the nuisance mask (CSF, WM, combined, or temporal). The remaining components are dropped from consideration. The head‐motion estimates calculated in the correction step were also placed within the corresponding confounds file. The confound time series derived from head motion estimates and global signals were expanded with the inclusion of temporal derivatives and quadratic terms for each (Satterthwaite et al., 2013). Frames that exceeded a threshold of 0.5 mm FD or 1.5 standardised DVARS were annotated as motion outliers. All resamplings can be performed with *a single interpolation step* by composing all the pertinent transformations (i.e., head‐motion transform matrices, susceptibility distortion correction when available, and co‐registrations to anatomical and output spaces). Gridded (volumetric) resamplings were performed using ANTs, configured with Lanczos interpolation to minimise the smoothing effects of other kernels (Lanczos, 1964). Non‐gridded (surface) resamplings were performed using mri_vol2surf (FreeSurfer).

Many internal operations of *fMRIPrep* use *Nilearn* 0.5.2 (Abraham et al., 2014, RRID:SCR_001362), mostly within the functional processing workflow. For more details of the pipeline, see the section corresponding to workflows in *fMRIPrep*'s documentation.

#### 2.1.7 fMRI data analyses

First‐level and second‐level analyses were carried out using SPM12 (Wellcome Trust Centre for Neuroimaging, London) in MATLAB 2018b and R2019a (MathWorks, Natick, MA). Seven mental and nine pain events identified by Richardson et al. (2018) were coded for the analyses. These events were derived from a reverse correlation analysis replicated across two adult samples. Besides mental and pain events, predictors of no interest were included (FD, six head‐motion parameters, and a subset of the anatomical CompCor confounds (i.e., WM and CSF decompositions). For one participant, no T1w image was available and CompCor could not be estimated and included as predictors of no interest. The model parameters were set following the recommendations of Jacoby et al. (2016): standard haemodynamic response function; reference time‐bin: 8; high‐pass filtering (128 s per cycle); interactions were not modelled; global normalisation (scaling); serial correlations ignored). Images were masked with a GM mask (threshold: 0.8). Simple contrasts (mental > pain; pain > mental) were calculated and the resulting contrast images were smoothed (5 mm smoothing kernel). For the second‐level analyses, one‐sample *t* tests were used for each dataset (*p* < .001 uncorrected, *k* = 10, with an average GM mask applied). For the ROI analyses, contrast values were extracted from the six theory‐of‐mind regions (bilateral TPJ, precuneus, dorsomedial prefrontal cortex (dMPFC), middle medial prefrontal cortex (mMPFC), and ventromedial prefrontal cortex (vMPFC)) and seven pain matrix regions (anterior middle cingulate cortex and bilateral secondary somatosensory cortex (SII), insula, and bilateral middle frontal gyrus) using the MarsBaR toolbox (Brett, Anton, Valabregue, & Poline, 2002). Coordinates were derived from Richardson et al. (2018) and a 9 mm sphere was used for each region (Tables S5 and S6).

#### 2.1.8 Main analyses

To test the relationship between dispositional anthropomorphism and theory‐of‐mind activation, and to account for the exploratory nature of these analyses, we used Bayesian regression analyses. This approach allowed us to assess the strength of the relationship as well as assess the evidence for and against the null (no relationship). To test for the possibility of a linear *and* non‐linear relationship between dispositional anthropomorphism and theory‐of‐mind network activation, we specified a model with mental > pain contrast values as dependent variable and IDAQ scale scores as linear and quadratic predictors for each region‐of‐interest separately. All variables were centred and scaled before specifying and fitting the models. Given the exploratory nature of this analysis, we estimated the models with uninformative (default, flat) priors (Student's *t*‐distribution with mean of 0, 3 degrees of freedom and a scale of 10). A Gaussian distribution was fitted to the data, and we used four Markov chains with 4,000 iterations (and a warm‐up of 2,000 iterations). Besides an individual model for each region‐of‐interest, we fitted a model for all regions of the theory‐of‐mind network combined. A similar approach was employed for the regions of the pain matrix. The {brms} package (version 2.12.0) was used in R (version 3.6.3) and Stan (version 2.19.1) (Bürkner, 2017). All models converged, with Rhat values below 1.1. We report the posterior mean regression coefficient (*b*) with estimated error and 95% credible credibility interval. Following the procedure outlined by Kruschke (2018), we used a highest density interval (HDI) of the posterior distribution and a region of practical equivalence (ROPE) around the null decision rule. This approach uses the Bayesian posterior distributions for each predictor to help decide whether it is possible to accept or reject the null value. If the HDI of the predictor lies within the ROPE the null value will be accepted, while if the HDI lies outside of the ROPE the null value will be rejected, and if there the HDI does not completely lie inside or outside of the ROPE no decision can be made. Similar to the interpretation of *p*‐values and Bayes factors, we acknowledge that the HDI + ROPE method has its limitation. However, we deem the inclusion of a decision rule appropriate in the context of the overall research question as it provides a categorical decision that will benefit the reader while still considering the magnitude of the parameter and uncertainty thereof in contrast to *p*‐values and Bayes factors (Kruschke, 2018).

#### 2.1.9 Control analyses

One potential problem of the mental versus pain contrast used in the main analyses could be masking of the effect of interest, that is, anthropomorphism could modulate activity during events that trigger mentalising *and* during events that show the main characters experiencing pain. To further validate our results and conclusion, we re‐ran control analyses using the hand‐coded events from Jacoby et al. (2016). In addition to events triggering mentalising (four events) and empathy for pain (seven events), events of interest included scenes in which the film characters interacted with each other without triggering internal state prediction (social events; five events), and events unrelated to the main characters such as other birds flying (control events; three events). To counteract possible masking, we ran the following contrasts of interest: (a) mental versus control events; and (b) pain versus control events. We also included the following general contrasts: (c) social versus control events; and (d) mental + pain + social versus control events. Next, we ran similar Bayesian models for these contrasts separately to test the extent to which IDAQ scale scores (linearly or quadratically) predict the contrast estimates across all regions combined as well as for the six individual regions of the theory‐of‐mind network. As a separate task‐independent sensitivity control analysis, we extracted contrast values for the left TPJ and vMPFC based on the coordinates of two previous studies on anthropomorphism and the theory‐of‐mind network (Cullen et al., 2014; Waytz, Morewedge, et al., 2010).

### 2.2 Results and discussion

**FIGURE 1:**
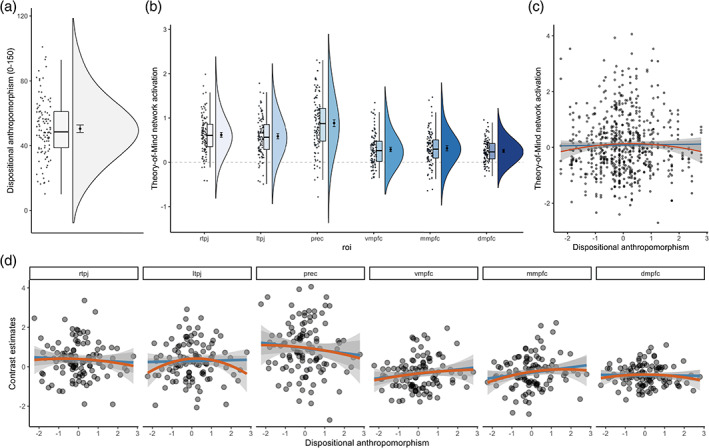
Dispositional anthropomorphism and theory‐of‐mind network activation. (a) Dispositional anthropomorphism across the sample as measured with the Individual Differences in Anthropomorphism Questionnaire, (b) activation in the six regions of the theory‐of‐mind network during the observation of scenes that trigger mentalising compared to scenes that trigger pain perception during an animated film, no clear relationship between dispositional anthropomorphism and activity (c) across the theory‐of‐mind network and (d) within the individual regions (quadratic predictor in red, linear predictor in blue). Indices are centred and scaled in (c) and (d). dmpfc, dorsomedial prefrontal cortex; mmpfc, middle medial prefrontal cortex; prec, precuneus; rtpj and ltpj, right and left temporoparietal junction; vmpfc, ventromedial prefrontal cortex

**FIGURE 2:**
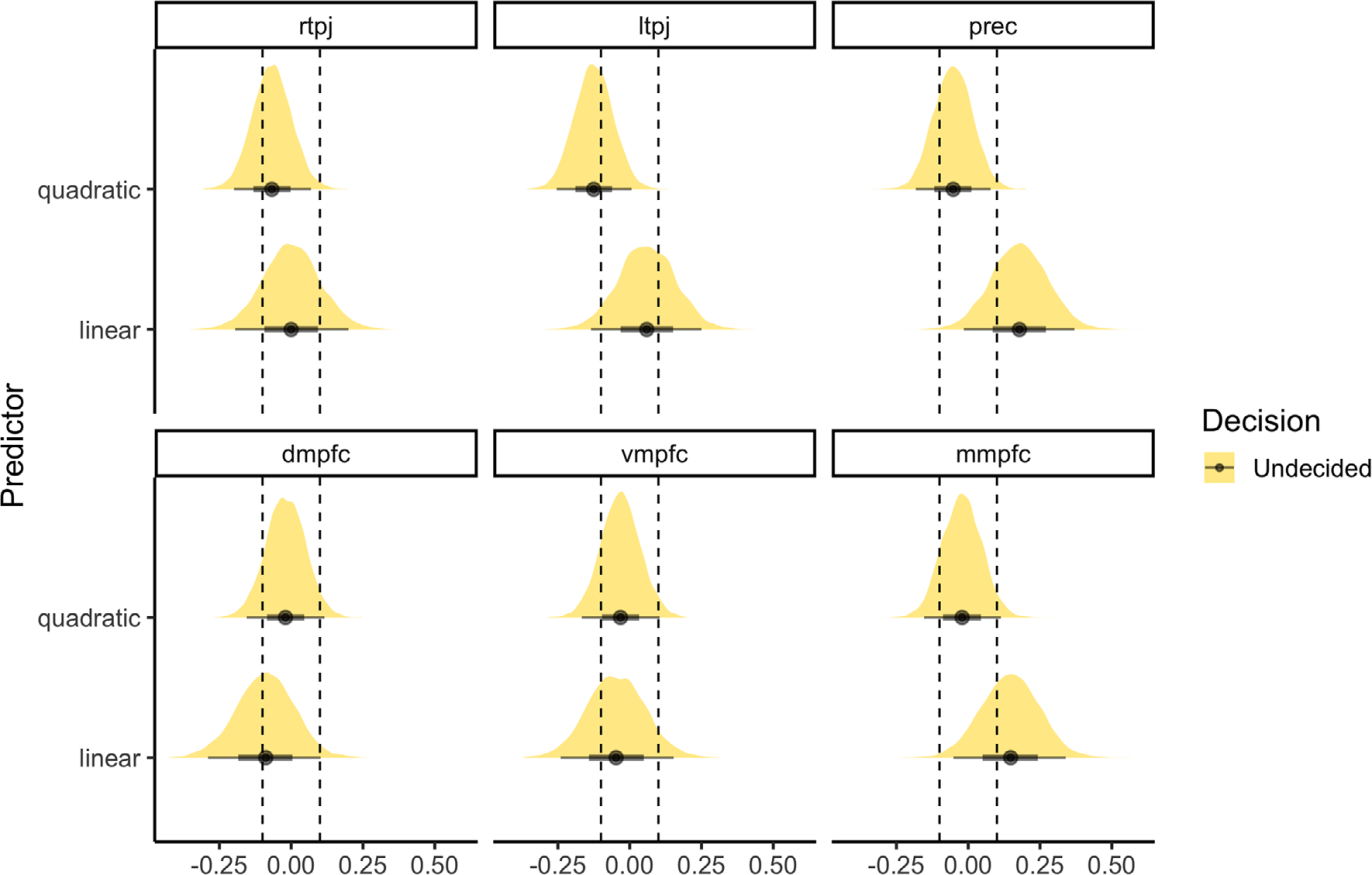
Posterior distribution for the linear and quadratic dispositional anthropomorphism predictor for each region of the theory‐of‐mind network in Experiment 1. The highest density interval of the posterior distribution and a region of practical equivalence around the null decision rule suggest that for all regions of the theory‐of‐mind network the null could be not be accepted or rejected for the linear and quadratic dispositional anthropomorphism predictor. dmpfc, dorsomedial prefrontal cortex; mmpfc, middle medial prefrontal cortex; prec, precuneus; rtpj and ltpj, right and left temporoparietal junction; vmpfc, ventromedial prefrontal cortex

**FIGURE 3:**
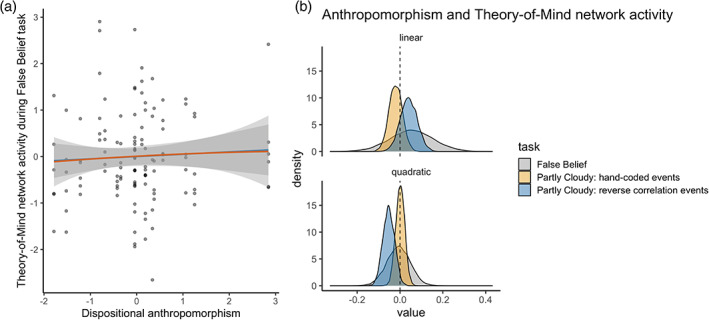
Dispositional anthropomorphism does not modulate theory‐of‐mind network activation across diverse measures. (a) No clear relationship between dispositional anthropomorphism and activity across the theory‐of‐mind network when participants read stories containing false beliefs compared to false depictions of photographs. (b) Posterior distributions for the linear and quadratic dispositional anthropomorphism predictor for each task measure of theory‐of‐mind network activity. Dispositional anthropomorphism did not modulate activity in the theory‐of‐mind network during passive viewing of an animated movie or during the false belief task. Contrast used per task: false belief versus false photograph stories (false belief task;1 Dodell‐Feder, Koster‐Hale, Bedny, & Saxe, 2011), scenes that trigger mentalising versus control events unrelated to the main characters (Partly Cloudy: hand‐coded events; Jacoby et al., 2016), and scenes that trigger mentalising versus scenes that trigger empathy for pain (Partly Cloudy: reverse correlation; Richardson et al., 2018)

The median disposition of participants in our sample to anthropomorphise was 48.5 (on scale from 0 to 150) with an interquartile range (IQR) of 22.25, and a minimum score of 10 and a maximum score of 101 (Figures 1a and S1). Whole‐brain and region‐of‐interest analyses showed robust activation for mental events compared to pain events across the theory‐of‐mind network, and for the inverse contrast in the pain matrix (Figures 1b and S2–S4). Dispositional anthropomorphism was not a consistent predictor of activity across the theory‐of‐mind network (Figure 1c). The estimated posterior regression coefficient for the linear predictor was 0.04, 95% credibility interval [−0.04 to 0.12], and −0.05 [−0.11 to 0] for the quadratic predictor. An in‐depth look at individual regions of the theory‐of‐mind network showed that dispositional anthropomorphism scores did not predict activity in these regions (Figure 1d). The estimated posterior regression coefficients for both the linear and quadratic predictor varied across regions (Figure 2). While dispositional anthropomorphism scores were weakly predictive of activity in the left TPJ, with a posterior regression coefficient of −0.13 [−0.25 to 0.01] for the quadratic predictor, other regions did not show similar effects (Table 1, Figure S5). For all predictors 0 was included in the 95% credibility interval. While the HDI of the posterior distribution overlapped with the ROPE, the data did not provide conclusive evidence on the presence or absence of a relationship between dispositional anthropomorphism and theory‐of‐mind network activity (Figure 2). In contrast to the theory‐of‐mind network, the null value could be accepted for the regions of the pain matrix (Figures S6–S8, Table S7). Control analyses revealed that regardless of the type of contrast used, for example, mental versus pain or mental versus control, dispositional anthropomorphism did not modulate theory‐of‐mind network activity (Figure 3, Table S8, Figures S9 and S10). Similarly, task‐independent regions derived from previous reports on the association of anthropomorphism and the theory‐of‐mind network also did not show a relationship between these forms of mind perception (Table S9).

**TABLE 1:** Estimated posterior regression coefficient for the linear and quadratic anthropomorphism predictor across each theory‐of‐mind network region

	rtpj		ltpj		prec		vmpfc		mmpfc		dmpfc	
Predictors	Estimates	CI (95%)	Estimates	CI (95%)	Estimates	CI (95%)	Estimates	CI (95%)	Estimates	CI (95%)	Estimates	CI (95%)
Intercept	0.03	−0.20 to 0.27	0.13	−0.11 to 0.35	0.02	−0.21 to 0.25	0.02	−0.21 to 0.26	0.05	−0.17 to 0.28	0.07	−0.17 to 0.30
Linear predictor	−0.05	−0.24 to 0.15	0.06	−0.13 to 0.25	−0.09	−0.29 to 0.10	0.15	−0.05 to 0.34	0.18	−0.02 to 0.37	−0.00	−0.20 to 0.20
Quadratic predictor	−0.03	−0.17 to 0.10	−0.13	−0.25 to 0.01	−0.02	−0.15 to 0.12	−0.02	−0.15 to 0.11	−0.05	−0.18 to 0.08	−0.07	−0.20 to 0.07
*R*^2^ Bayes	.017		.042		.021		.031		.041		.021	

CI (95), 95% credibility interval; dmpfc, dorsomedial prefrontal cortex; mmpfc, middle medial prefrontal cortex; prec, precuneus; rtpj and ltpj, right and left temporoparietal junction; vmpfc, ventromedial prefrontal cortex.

These findings provide good evidence that there is no clear or obvious positive relationship between an individual's tendency to anthropomorphise and activity in the theory‐of‐mind network. Upon closer inspection, the literature does not provide a clear picture either. A recent behavioural study found no relationship between measures of dispositional anthropomorphism and direct and indirect measures of theory‐of‐mind in children and adults (Tahiroglu & Taylor, 2019). In the same study, no clear relationship between situational (e.g., task‐based) anthropomorphism and theory‐of‐mind was observed. In contrast with these behavioural observations, several neuroimaging studies found an association between situational measures of anthropomorphism and activity in regions of the theory‐of‐mind network (Chaminade et al., 2007; Waytz, Morewedge, et al., 2010). As theory‐of‐mind describes a way to understand and predict another agent's behaviour, anthropomorphism can serve as one way to achieve this (Waytz, Morewedge, et al., 2010). Anthropomorphising non‐human agents could help in improving prediction of the behaviour of these agents, similar to extracting invariance (e.g., emotions) out of variance (e.g., behaviour, movement; Heider, 1958). If so, the overlap and association between anthropomorphism and theory‐of‐mind would be stronger at the level of the situation (e.g., task‐based). At this level, it can be expected that anthropomorphism facilitates theory‐of‐mind in a straightforward, linear fashion. A higher need to understand or predict the situation of a non‐human agent would require more attribution of human‐like characteristics (Waytz, Cacioppo, & Epley, 2010a; Waytz, Gray, et al., 2010; Waytz, Morewedge, et al., 2010). However, at the personality level, a more complex relationship might be possible.

Exploratory data analysis in Experiment 1 led us to believe that perhaps the relationship could be better described as a quadratic relationship, rather than the expected linear relationship. One possible reason for such a relationship is that people mostly rely on theory‐of‐mind for situations in which there is ambiguity in interpretation. In other words, it is possible that those who fall in the middle of the scale for dispositional anthropomorphism rely more heavily on theory‐of‐mind in situations that have potential for anthropomorphism (e.g., during the animated film). Individuals with lower levels of dispositional anthropomorphism are less likely to use theory‐of‐mind as they rarely engage in anthropomorphising non‐human agents, while individuals with higher levels of dispositional anthropomorphism readily and efficiently perceive and understand the behaviour of these agents, thereby requiring no explicit theory‐of‐mind. Thus, it is likely that the strength and type of relationship is dependent on the level of analysis (situational vs. dispositional).

To explore these questions further, we tested the relationship between measures of theory‐of‐mind and dispositional *and* situational anthropomorphism in a follow up preregistered experiment. While viewing the same short animation as in Experiment 1, participants provided ratings on their belief of the capacities of the film characters, which served as measures of situational anthropomorphism. In order to establish a link between anthropomorphism and theory‐of‐mind at the situational level, we approximated the participant's ability to understand and predict the behaviour of the characters throughout the movie. These latter effectance ratings served as a proxy of theory‐of‐mind and are related to a process of understanding and predicting uncertainties in the environment, for example, the internal and behavioural states of an agent (Waytz, Morewedge, et al., 2010; White, 1959). Besides dispositional anthropomorphism, we also measured performance on a false belief task to test if a potential link with anthropomorphism generalises across measures of theory‐of‐mind. As in Experiment 1, we tested for both linear and non‐linear (cf. quadratic) effects. Based on the findings of Experiment 1 and pilot data (*n* = 20), we hypothesised: (a) a linear and positive relationship between situational anthropomorphism and theory‐of‐mind; (b) a quadratic relationship between dispositional anthropomorphism and theory‐of‐mind; and we expected that (c) situational anthropomorphism would be a better predictor of theory‐of‐mind than dispositional anthropomorphism.

## 3 EXPERIMENT 2

### 3.1 Materials and methods

#### 3.1.1 Preregistration and data statement

The data used for Experiment 2 were collected online, and the OSF preregistration can be found at https://osf.io/tuq4a. We report all measures in the study, all manipulations, any data exclusions and the sample size determination rule.

#### 3.1.2 Participants

A representative sample from the United Kingdom was recruited through Prolific (www.prolific.co) stratified across age, sex, and ethnicity based on UK Office of National Statistics census data. Our target sample size was 320 participants, to account for potential exclusion or missing data. Simulations indicate that robust estimates are obtained when *n* > 200 (Schönbrodt & Perugini, 2013). We overshot our target sample, 333 participants completed parts of the experiment, with 311 of those participants completing everything. The sample consisted of 154 women and 157 men, aged between 18 and 27 (*n* = 49), 28–37 (*n* = 57), 38–47 (*n* = 60), 48–57 (*n* = 52), and 58 or older (*n* = 93) of Asian (*n* = 27), Black (*n* = 20), Mixed (*n* = 13), White (*n* = 241), or another ethnicity (*n* = 10) (Figure S11). Participants provided informed consent, were naive to the goal of the study, and received a debriefing and compensation of £3 upon completion. The study procedure was approved by the Research Ethics Committee of the College of Science and Engineering at the University of Glasgow (protocol number: 300190004).

#### 3.1.3 Rating task

To measure situational anthropomorphism and effectance, participants viewed the same animated film “Partly Cloudy” as in Experiment 1 and rated 16 short scenes throughout the film. The same seven “mentalising/theory‐of‐mind” and nine “pain” triggering events as in Experiment 1 were used. Participants rated both main characters, the stork, Peck (Scenes 6–13, and 15–16) and the cloud, Gus (Scenes 4–16), as well as two support characters, nameless cloud and nameless stork (Scenes 1–3). The characters were not referred to by their name, but by using “the cloud” and “the stork.” After each of the 16 “mental” or “pain” scenes, the film was paused, and participants were asked to provide ratings on situational anthropomorphism and effectance. For the situational anthropomorphism ratings, participants rated the extent to which they believed the characters of the film (a stork and a cloud) possessed certain capacities. Participants rated if the character is able to “*choose and control its own actions*,” “*aware of itself and its thoughts and feelings*,” “*do what it wants*,” and has “*preferences and plans*” and “*feelings*” on a scale from 1 (not at all) to 7 (very much). These items correspond to the definitions used by Waytz, Morewedge, et al. (2010), and Waytz et al. (2018) for “*free will*,” “*consciousness*,” “*a mind of its own*,” “*intentions*,” and “*experience emotions*.” For the effectance ratings, participants indicated the extent to which they felt capable of “*imagining what the cloud (or stork) will do next”* and “*thinking about what the cloud (or stork) is doing and why*” on a scale from 1 (not at all) to 7 (very much). These items correspond to the definitions used for “*understood the character*” and “*feel capable of predicting its future behaviour*” in the study by Waytz, Morewedge, et al. (2010). For these latter two items, a 7‐point scale instead of a 10‐point scale was used to allow for presentation alongside the situational anthropomorphism items. The order in which the anthropomorphism and effectance rating items were displayed during the rating task was randomised for each participant. The focus of the rating was randomised across participants, with some participants asked to give ratings for the cloud first and the stork second, while other participants were asked to rate the stork first and the cloud second. After 4:02 min, the film was paused, and participants were asked to describe in one or two sentences what they think would happen next (to a maximum of 2,000 characters including spaces). This served as an attention check and an exploratory measure of theory‐of‐mind. After viewing the film, participants were asked to indicate if they had seen the film before (yes/no). Eighteen out of 311 participants had seen the movie before.

#### 3.1.4 False belief

In the false belief task (Dodell‐Feder et al., 2011), participants were asked to read 10 false‐belief stories, detailing incorrect beliefs about the world held by characters, and false‐photograph stories, detailing outdated photographs, maps or signs of the world. Each story was followed by a statement about the story. Participants were asked to indicate if the statement about the false‐belief or false‐photograph story was true (1) or false (2). The stories were presented for 15 s, followed by the statements which were presented for 6 s (Spunt et al., 2015).

This false belief task has previously been shown to robustly activate the theory‐of‐mind network (Dodell‐Feder et al., 2011). Both the false belief task and the Partly Cloudy film used in Experiment 1 have been shown to activate similar regions (Jacoby et al., 2016), suggesting convergence in the use of activity during these tasks as a proxy for theory‐of‐mind. While the task has been used as an exploratory behavioural measure of theory‐of‐mind (Spunt et al., 2015), it has not been validated as a measure of false‐belief reasoning ability per se. To provide further validation and justification for this task as a measure of general theory‐of‐mind, we reanalysed data from two studies (Darda, Butler, & Ramsey, 2018, Darda & Ramsey, in preparation). Participants (*n* = 86) completed the false belief task to localise the theory‐of‐mind network while behavioural indices (accuracy and reaction times) were recorded. Results indicate that accuracy and response times on the false belief trials of the task were consistently associated with activity within and across the theory‐of‐mind network during this task. Accuracy on the false belief trials was positively correlated to averaged theory‐of‐mind network activity, Pearson's *r*(84) = 0.43, 95% confidence interval [0.24–0.59], while response times on these trials were negatively correlated with activity in this network, *r*(84) = −0.30 [−0.09 to −0.48]. These patterns were also observed for activity within the individual regions of the network (Figure S12, Table S10, Supplementary Results). These neuroimaging results suggest that the false belief task can be used as a proxy of general theory‐of‐mind, and that this is reflected in behavioural measures (i.e., false belief accuracy). Twenty of the 86 participants also completed the IDAQ. This allowed us to explore the relationship between dispositional anthropomorphism and activity across regions of the theory‐of‐mind network during the false belief task. Specifically, we fitted a similar Bayesian regression model as in Experiment 1, with false belief > false photograph contrast values as the dependent variable and IDAQ scale scores as linear and quadratic predictors (number of observations: 120). Consistent with the findings for activation in the theory‐of‐mind network during passive viewing of the Partly Cloudy animated film, dispositional anthropomorphism did not consistently predict activity across the theory‐of‐mind network when participants engaged in a false belief task (Figure 3). The estimated posterior regression coefficient for the linear predictor was 0.05, [−0.15 to 0.25], and −0.01 [−0.11 to 0.10] for the quadratic predictor.

#### 3.1.5 Procedure

Besides the rating task and the false belief task, participants completed the same dispositional anthropomorphism questionnaire as in Experiment 1 (IDAQ; Waytz, Cacioppo, & Epley, 2010a). The order in which participants completed the parts of the experiment was randomised, with some participants completing the rating task and false belief task first, and the IDAQ questionnaire second, while other participants completed the parts in the opposite order. At the end of the experiment, participants answered a final question on how often they engaged with robots as part of a different unrelated research project. All parts of this experiment were completed through Pavlovia (https://pavlovia.org/; Peirce et al., 2019), in order to allow flexibility in the order of completion. The total experiment took 20–30 min.

#### 3.1.6 Data processing

The five anthropomorphism items were averaged to create a situational anthropomorphism score, while the two effectance items were averaged to create an effectance score (Waytz, Cacioppo, & Epley, 2010a, ; Waytz, Gray, et al., 2010; Waytz, Morewedge, et al., 2010). Accuracies (percentage correct) for the false‐belief and false‐photograph stories were calculated separately (Spunt et al., 2015), and the responses for the IDAQ and IDAQ‐NA scale were summed separately (Waytz, Cacioppo, & Epley, 2010a). All indices were centred and scaled before analyses.

The following exclusion criteria were specified in the preregistration: participants that failed the attention check during the film (no characters typed, *n* = 13), who showed no variability in their ratings (*SD* of <0.5, *n* = 20), who clicked through the rating task (mean response duration <1 s, *n* = 10) or IDAQ (duration <1 min, no participants), and with an accuracy <50% on the false belief trials of the false belief task (*n* = 29) were excluded. Furthermore, participants who did not complete all aspects of the online task (*n* = 22) or who had ≥5 missing responses for the false belief task (*n* = 9) were excluded. Final *n* for analyses is 241, with 92 participants excluded.

#### 3.1.7 Preregistered analyses

We preregistered a Bayesian multivariate regression approach to test a linear and non‐linear (cf. quadratic) relationship between situational and dispositional anthropomorphism and theory‐of‐mind. We used a skew‐normal distribution model, as visualising the data showed the data was not symmetric and normally distributed (Martin & Williams, 2017). Anthropomorphism ratings during the film and IDAQ score served as linear and quadratic predictors of effectance ratings during the film and false belief accuracy. The models were estimated with weakly informative priors, normal (0,1), which avoids inappropriate inferences that can be the result when using non‐informative priors, without supplying strict information (Gelman, Jakulin, Pittau, & Su, 2008). A skewed‐normal distribution was fitted for both the effectance and false belief sub‐models, and four Markov chains with 4,000 iterations (and a warm‐up of 2,000 iterations) were used.

Besides the full model, including all variables, we also specified the model incrementally. Starting with an intercept‐only model, we then added the predictors in a stepwise fashion following the three hypotheses: a first model for Hypothesis 1 with only a linear situational anthropomorphism predictor, a second model for Hypothesis 2 with only a quadratic dispositional anthropomorphism predictor, and a third model for Hypothesis 3 with both a linear situational and quadratic dispositional anthropomorphism predictor. Models were specified in the {brms} package (version 2.12.0) in R (version 3.6.3) with Stan (version 2.19.1) (Bürkner, 2017). All models converged, with Rhat values below 1.1. We used approximate leave‐one‐out cross‐validation based on the posterior likelihood to compare these models and establish the model with the best fit using the {loo} package (version 2.2.0) in R (Vehtari, Gelman, & Gabry, 2017). We estimated the leave‐one‐out information criteria for each model, with smaller values indicating a better fit, and calculated the differences between these estimates of the different models. In addition, similar to Experiment 1, we used the HDI of the posterior distribution and a ROPE around the null decision rule (Kruschke, 2018).

#### 3.1.8 Exploratory analyses

To further map the relationship between different forms of mind perception, we tested the extent to which anthropomorphism was related to actual predictions made by participants during the video. The moment the movie was paused corresponded to an event where one of the main characters (the stork) flies away to another cloud leaving room for several explanations. An example of a correct prediction would be that the stork seeks help of the other cloud and will return to the original cloud to continue their work, while an incorrect prediction would be that the stork leaves the cloud to never return. Two coders (R. H. and M. K.) rated the prediction on accuracy, on a scale from 0 (incorrect) to 100 (correct), as well as made a binary decision (correct or incorrect) and rated the extent to which the prediction relied on/contained theory‐of‐mind, on a scale from 0 (very little) to 100 (very much). Predictions made by the participants were randomly presented to the coders using Gorilla (Anwyl‐Irvine, Massonnié, Flitton, Kirkham, & Evershed, 2020). Besides applying the preregistered exclusion criteria, participants that already were already familiar with the film were removed (*n* = 14) and one additional participant was excluded because they did not provide a prediction (but did not fail the attention check). Final *n* for the exploratory analyses was 226. Both the rating of accuracy and theory‐of‐mind were highly correlated between the two coders, accuracy: *r*(226) = 0.76, 95% confidence interval [0.70, 0.81] and theory‐of‐mind: *r*(226) = 0.76, 95% confidence interval [0.70, 0.81]. Discrepancies, that is, one of two coders made a different binary decision, were resolved through discussion. For both the accuracy and theory‐of‐mind of the prediction, the ratings were averaged across the coders, centred and scaled. A Bayesian regression model was specified for accuracy ratings and theory‐of‐mind ratings separately with linear and quadratic situational and dispositional anthropomorphism predictors. The skewed‐normal distribution models were estimated with weakly informative priors, normal (0,1).

### 3.2 Results and discussion

**FIGURE 4:**
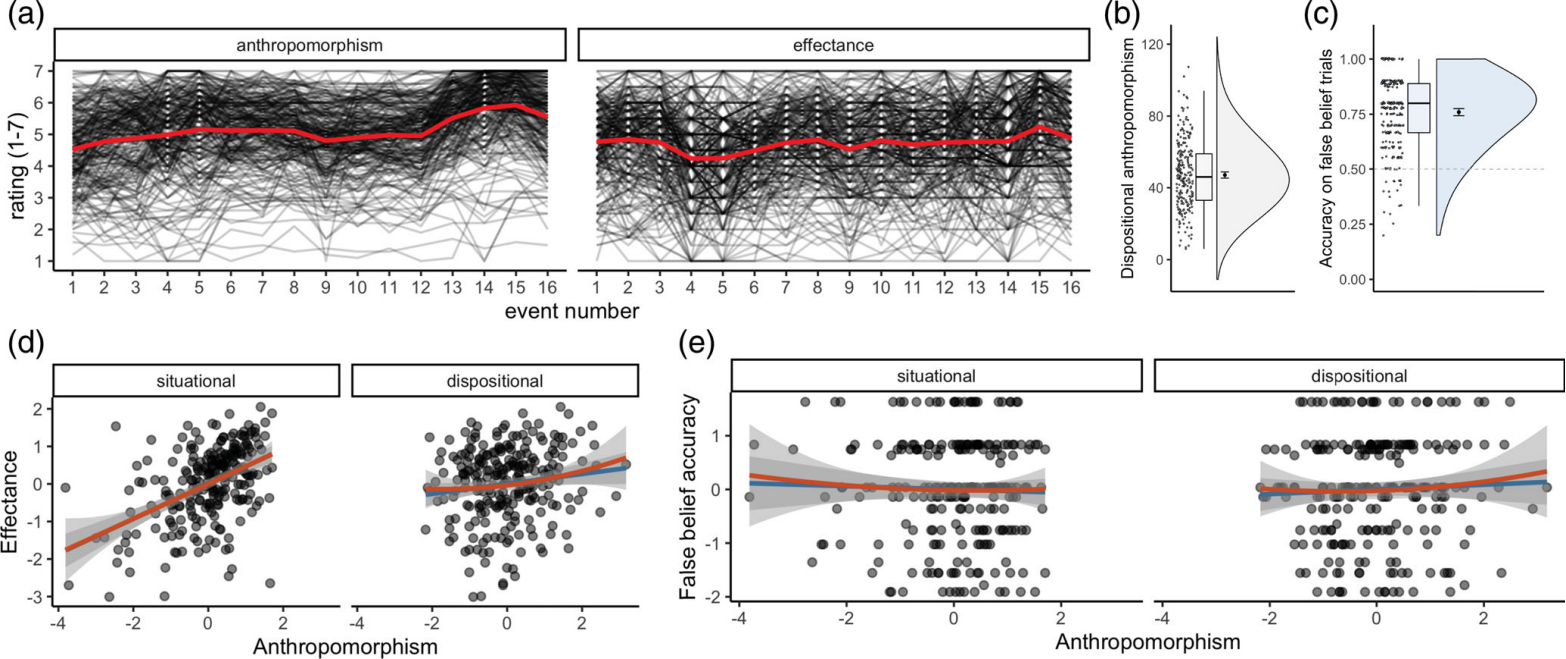
Situational and dispositional anthropomorphism and theory‐of‐mind. (a) Situational anthropomorphism and effectance ratings, a proxy of theory‐of‐mind, fluctuated throughout the film (average ratings are shown in red and individual participants are represented by black lines), (b) dispositional anthropomorphism across the sample, (c) performance for the false belief trials, a proxy of general theory‐of‐mind, of the false belief task (dashed line indicates preregistered exclusion criteria of <.5), (d) situational, but not dispositional anthropomorphism linearly predicted effectance ratings, (e) neither situational nor dispositional anthropomorphism predicted false belief accuracy (quadratic predictor in red, linear predictor in blue). Indices are centred and scaled in panels (d) and (e)

Situational anthropomorphism and effectance ratings fluctuated throughout the film but showed distinct patterns (Figure 4a). A gradual increase in situational anthropomorphism ratings was observed as the film progressed and the characters became more familiar to the viewer (average rating across the film: 5.11, 95% confidence interval [5.09–5.14], first event: 4.53 [4.46–4.57], last event: 5.55 [5.50–5.61], Cronbach's *α* = .9838 [.9799–.9873]). Effectance ratings did not show a gradual increase (average rating across the film: 4.74 [4.70–4.78], first event: 4.77 [4.65–4.88], last event: 4.88 [4.78–4.98], Cronbach's *α* = .9626 [.9535–.9707]), but reflected event‐by‐event fluctuations and were sensitive to distinct events in the film (e.g., introduction of new characters, revelation of intent of the main character). The mean disposition to anthropomorphise was 46 with an IQR of 26 (Cronbach's *α* = .78 [.75–.82]), and a minimum score of 6 and a maximum score of 102 (Figure 4b). Mean accuracy on the false belief task was 0.8 with an IQR of 0.2 (Figure 4c). While there was a positive correlation between the anthropomorphism indices, *r*(239) = 0.30, [0.18–0.41], the effectance ratings and false belief accuracy indices showed no such relation, *r*(239) = 0.01, [−0.12 to 0.13] (Figure S13).

Results from the Bayesian multivariate regression analysis showed that situational anthropomorphism ratings, but not dispositional anthropomorphism, predicted effectance ratings (Figure 4d). Specially, only linear situational anthropomorphism ratings, with an estimated posterior regression coefficient of 0.45, 95% credibility interval [0.31–0.59], predicted effectance ratings. Neither quadratic situational anthropomorphism ratings, 0.03 [−0.04 to 0.10], nor dispositional anthropomorphism, linear: −0.03 [−0.14 to 0.09], quadratic: 0.07 [−0.01 to 0.16], predicted effectance ratings. No relation was found between anthropomorphism indices and false belief accuracy (Figure 4e). Neither situational, −0.04 [−0.20 to 0.11], quadratic predictor: 0.01 [−0.07 to 0.10], or dispositional anthropomorphism, linear predictor: 0.06 [−0.08 to 0.19], quadratic predictor: 0.02 [−0.07 to 0.11], predicted performance on the false belief task (Table 2).

**TABLE 2:** Estimated posterior regression coefficient for each predictor for both theory‐of‐mind indices

	Effectance		False belief	
Predictors	Estimates	CI (95%)	Estimates	CI (95%)
Intercept	−0.10	−0.26 to 0.04	−0.02	−0.20 to 0.16
Linear situational	0.45	0.31– to 0.59	−0.04	−0.20 to 0.11
Quadratic situational	0.03	−0.04 to 0.10	0.01	−0.07 to 0.10
Linear dispositional	−0.03	−0.14 to 0.09	0.06	−0.08 to 0.19
Quadratic dispositional	0.07	−0.01 to 0.16	0.02	−0.07 to 0.11

CI (95): 95% credibility interval.

The importance of situational anthropomorphism ratings was also reflected in the results obtained from the leave‐one‐out cross‐validation based on the posterior likelihood. The first model, with only a linear situational anthropomorphism predictor (Hypothesis 1), provided the best fit for the data (Table S11, Figure S14). Compared to this situational anthropomorphism only model, a model with only a quadratic dispositional anthropomorphism predictor (Hypothesis 2) or a model with both a linear situational and quadratic dispositional anthropomorphism predictor (Hypothesis 3) provided a worse fit or did not improve the fit, respectively.

**FIGURE 5:**
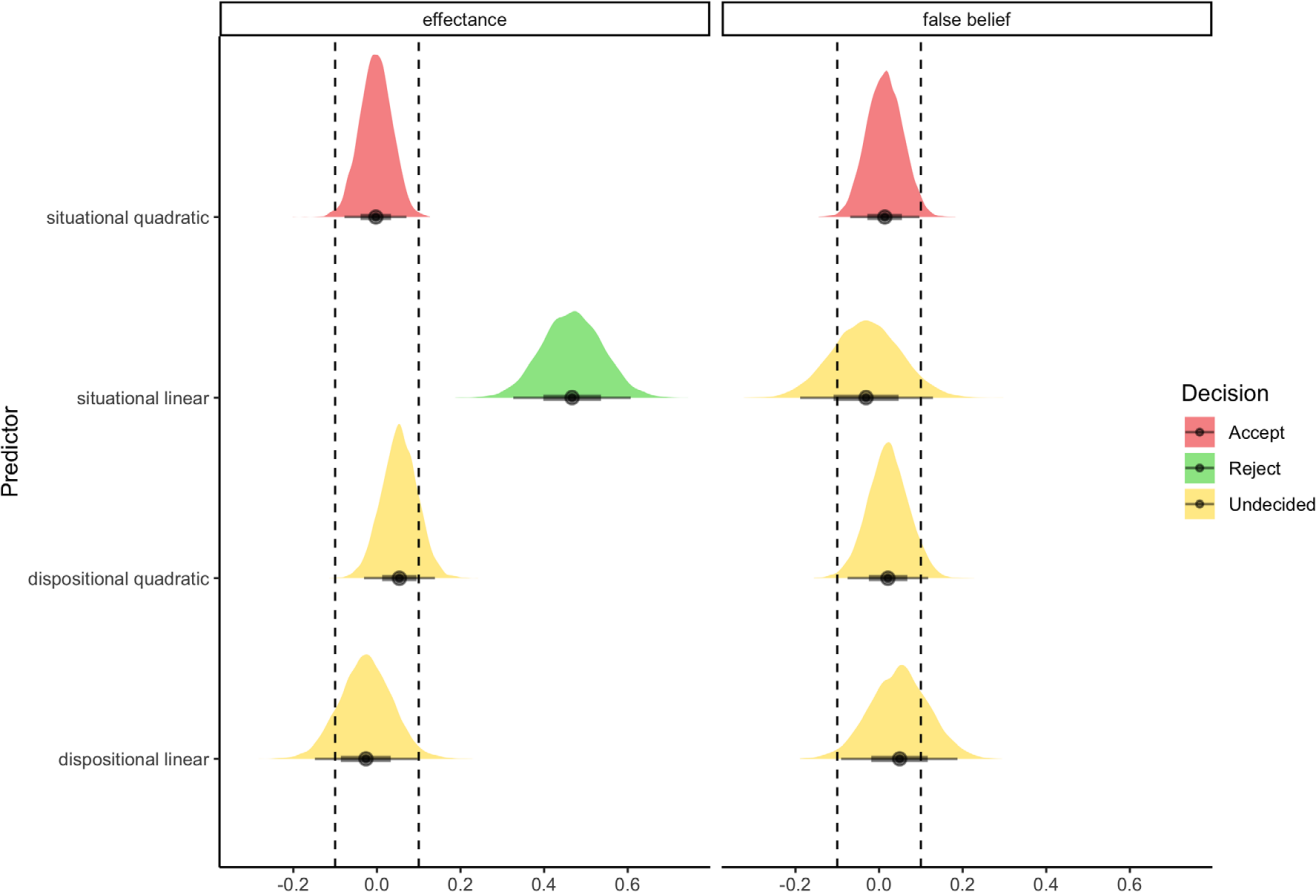
Posterior distribution for each predictor for both theory‐of‐mind indices in Experiment 2. The HDI of the posterior distribution and a region of practical equivalence around the null decision rule suggest that for effectance ratings, the null could be rejected for the linear situational anthropomorphism predictor and accepted for the quadratic situational anthropomorphism predictor, while for false belief accuracy only the null could be rejected for the quadratic situational anthropomorphism predictor

For all predictors other than the linear situational anthropomorphism predictor for effectance ratings, 0 was included in the 95% credibility interval (Figure 5). The null value could be accepted for the quadratic situational anthropomorphism predictor of both effectance ratings and false belief performance, while the null value could be rejected for the situational anthropomorphism predictor of effectance ratings. While the HDI of the posterior distribution overlapped with the ROPE for the linear and quadratic dispositional anthropomorphism predictors, the data did not provide conclusive evidence on the presence or absence of a relationship between dispositional anthropomorphism and theory‐of‐mind indices. Similar results were obtained when including the participants that did not meet the exclusion criteria or when excluding participants who were familiar with the film (Figures S15 and S16).

The effectance ratings might partly reflect participants' subjective evaluation of their ability to understand and predict the minds of the characters in the film. To circumvent this possibility, we tested the relationship between anthropomorphism and the accuracy of the actual open‐ended prediction made by the participants during the film and reliance on theory‐of‐mind. The average rated accuracy of the prediction was 33.57 [29.04–38.10], with 75 participants making a correct prediction and 151 participants making an incorrect prediction. The average rated reliance on theory‐of‐mind was 53.49 [49.28–57.70]. Accuracy of the prediction was correlated with the reliance on theory‐of‐mind, *r*(226) = 0.40 [0.28–0.50] and increased reliance on theory‐of‐mind was observed for participants that made a correct prediction, average rated reliance on theory‐of‐mind: 68.05 [62.61–73.49], compared to participants who made an incorrect prediction, 46.26 [40.89–51.63]. A weak but positive relationship was observed between these exploratory measures of theory‐of‐mind and situational and general measures of theory‐of‐mind (Table S12). Providing further support for the dissociation between anthropomorphism and theory‐of‐mind, results showed that neither situational anthropomorphism nor dispositional anthropomorphism was related to the accuracy of the prediction or the reliance of the prediction on theory‐of‐mind (Figure S17). While situational anthropomorphism linearly predicted the self‐rated ability to understand and predict the behaviour of the film characters, it did not predict the accuracy of the actual prediction made during the film, 0.00 [−0.06 to 0.07] or the reliance on theory‐of‐mind of this prediction, 0.02 [−0.08 to 0.12] (Table S13).

As predicted, a linear and positive relationship between situational anthropomorphism ratings and a measure of theory‐of‐mind, effectance ratings, was observed. As no relationship was observed between situational anthropomorphism ratings and the measure of general theory‐of‐mind, performance on the false belief task, these results provide only provide partial support for Hypothesis 1. Similarly, only partial support for Hypothesis 3 was found. Situational anthropomorphism ratings predicted effectance ratings better than dispositional anthropomorphism. No quadratic relationship between dispositional anthropomorphism and theory‐of‐mind indices was found, providing no support for the Hypothesis 2. Finally, exploratory analysis showed that anthropomorphism was unrelated to actual predictions made by participants.

It remains difficult to determine whether a true relationship exists between anthropomorphism and theory‐of‐mind. While situational anthropomorphism was a strong predictor of an individual's ability to understand and predict the characters' behaviour during the film, these effectance ratings can only be viewed as a proxy of situational theory‐of‐mind. Neither dispositional nor situational measures of anthropomorphism were predictors of general theory‐of‐mind, which was measured here as performance on the false belief task. Therefore, we do not claim that these findings are suggestive of a strong relationship between anthropomorphism and measures of theory‐of‐mind. The linear and quadratic situational and dispositional anthropomorphism predictors differed in their predictive power. Taken at face value, these results suggest that if there is a relationship between these constructs, this only holds at the situational level in a linear fashion.

## 4 GENERAL DISCUSSION

In the present study, we aimed to further examine the relationship between theory‐of‐mind and anthropomorphism. Using a multi‐method approach, we tested if situational and dispositional measures of anthropomorphism are predictive of behavioural and brain indices of theory‐of‐mind while watching an animated film. Across two experiments, we find no evidence for a clear relationship between dispositional measures of anthropomorphism and theory‐of‐mind. Only situational anthropomorphism was related to the ability to understand and predict the behaviour of the characters during the film, but not to classic measures of theory‐of‐mind in more general contexts. If a relationship between theory‐of‐mind and anthropomorphism exists, it appears to be more complex than initially thought, making it difficult to tease apart experimentally. Our results suggest that anthropomorphism cannot be considered a mere extension or analogue of theory‐of‐mind. We surmise that attributing a mind to artificial agents is something at least partly different from inferring hidden mental states of fellow humans.

Results from Experiment 1 provided inconclusive evidence as to whether dispositional anthropomorphism modulates activity in regions of the theory‐of‐mind network. Similar to some previous literature (Chaminade et al., 2007; Cullen et al., 2014), we saw slightly more evidence for a relationship between dispositional anthropomorphism and activity in one region of the theory‐of‐mind network, the left TPJ. Both functional (Chaminade et al., 2007) and structural (Cullen et al., 2014) associations between this region and dispositional anthropomorphism have been reported. There is some indication that the left and right TPJ might underpin context‐dependent decisions related to the social nature of an agent (Carter, Bowling, Reeck, & Huettel, 2012; Hortensius & Cross, 2018). However, as the null could not be rejected or accepted, the present result should be interpreted with caution. Similarly, the left and right TPJ have been implicated in not only theory‐of‐mind and related processes, but also in other more domain‐general functions (Darda et al., 2018). While in Experiment 1, we focussed on the theory‐of‐mind network, functional regions outside of this network, such as the fusiform face area, have been implicated in anthropomorphism (Kühn, Brick, Müller, & Gallinat, 2014). While univariate analysis showed that network activity did not capture variation in anthropomorphism, other analytic approaches such as functional connectivity and representational similarity analyses might provide different results. To fully capture the anthropomorphic perception of non‐human agents and objects it is important to look beyond brain regions implicated in theory‐of‐mind or other social cognitive processes, and map activity across diverse and possibly non‐social functional regions using different analytic tools (Henschel, Hortensius, & Cross, 2020).

Exploratory data analysis in Experiment 1 led us to believe that perhaps the relationship could be better described as a quadratic relationship, rather than the expected linear relationship. However, formal analyses in Experiment 2 did not find evidence for such a relationship. There was no clear evidence for a quadratic, or linear, link between dispositional anthropomorphism and the ability to understand and predict the behaviour of the characters of the film. This suggests that an individual's likelihood to perceive human states in non‐human agents or objects in everyday life does not strengthen their theory‐of‐mind. Situational anthropomorphism, on the other hand, predicted effectance ratings linearly. Individuals that attributed human features and mental states to the non‐human characters in the film indicated a better ability to understand and predict the behaviour of these characters. Experiment 2 enabled us to tease apart the broad concept of anthropomorphism in the hope of better understanding how situational and dispositional anthropomorphism differ. While situational and dispositional anthropomorphism were correlated in the current sample, they showed a different relationship with proxies of theory‐of‐mind. Taken at face value, it seems that dispositional anthropomorphism might not influence theory‐of‐mind‐like processes, but that situational anthropomorphism is one way to increase the prediction of hidden states of non‐human agents. Anthropomorphising the situation might help us to become more familiar, predict or master a situation, but people that have a higher tendency to anthropomorphise in general do not show a better understanding of a specific situation nor exhibit increased theory‐of‐mind. However, care is warranted in term of how generalisable this finding is, as the evidence on a relationship between situational anthropomorphism and theory‐of‐mind remains mixed at best (Tahiroglu & Taylor, 2019; Waytz, Morewedge, et al., 2010).

We approximated the multi‐dimensional construct of theory‐of‐mind using different measures. We acknowledge that these measures each have their strengths and weaknesses. A recent review that examined a number of classic theory‐of‐mind tasks found dramatic variability in what each task actually measures (François & Rossetti, 2020). In particular, the authors suggest that there is a lack of specificity when it comes to the terminology and measures of theory‐of‐mind. While, commonly, theory‐of‐mind is thought to describe the process of inferring others' hidden mental states, it appears that a number of classic assessments do not necessarily tap into mentalising capabilities. It may be that there is a dissociation between cognitive and affective theory‐of‐mind (Kalbe et al., 2010), For example, the false belief task is cognition‐dependent, with verbal intelligence influencing performance (Conway, Catmur, & Bird, 2019; Taylor & Carlson, 1997), and therefore might only be targeting the cognitive branch. This implies that different tasks can measure slightly different psychological constructs, from “perspective‐taking” to “empathy.” While these terms may converge in some situations, it appears that determining what theory‐of‐mind actually is, lies in an area of uncertainty and this is reflected in associated tasks.

A similar, more formal deconstruction of anthropomorphism can be achieved using terminology from the larger literature on mind perception (Fiske, Cuddy, Glick, & Xu, 2002; Gray, Gray, & Wegner, 2007; Haslam & Loughnan, 2014; Waytz, Gray, et al., 2010), and would be to distinguish two forms of anthropomorphism. The first form would be related to the perceived ability of an agent to act independently (also termed agency, human uniqueness, or competence), while the second form of anthropomorphism would be related to the perceived ability to feel (also termed experience, human nature, or warmth). As motivation influences the perception of these abilities (Waytz & Young, 2014), a beneficial avenue for future research would be to focus on more specific components of anthropomorphism and theory‐of‐mind using both behavioural and brain measures to comprehensively examine the link between these aspects of mind perception at the situational and individual level. For instance, one hypothesis based on our findings would be that there is a dissociation between affective theory‐of‐mind, as measured with effectance ratings, and cognitive theory‐of‐mind, as measured with false belief accuracy, when we anthropomorphise agents in terms of ability to act (“this character remembers”) and feel (“this character is angry”).

Alternatively, distinguishing different components of anthropomorphism would provide insight on processes beyond theory‐of‐mind that influence or underpin anthropomorphism. Evidence from both neuroimaging and behavioural studies points to the possibility that, at least for some aspects, anthropomorphism might be a low‐level process distinct from theory‐of‐mind. For instance, implicit anthropomorphism, measured by Kühn et al. (2014) as adjectives individuals used to describe cars that are also applicable to humans, was associated with activity in the fusiform face area and not in the TPJ and MPFC regions of the theory‐of‐mind network. While anthropomorphism is not the mere perception but also the attribution of human‐like characteristics to non‐human agents and objects, perception is an integral part of this process (Heider, 1958). When inferring the behavioural and internal states of a non‐human agent, one has to describe these states using labels. As suggested by Tahiroglu and Taylor (2019), language relevant to theory‐of‐mind could facilitate explaining and describing the behavioural and internal states of the observed agent. This, however, does not imply that theory‐of‐mind is necessary for anthropomorphism. Epley et al. (2007) distinguish between weak and strong forms of anthropomorphism, with the distinction that in the latter people truly believe that a non‐human agent has the ascribed characteristics while in the former people act as if the non‐human agent has the ascribed characteristics. Rather than being an active process underlying anthropomorphism, theory‐of‐mind could merely provide a way to describe the agent or situation (Tahiroglu & Taylor, 2019).

Observing, inferring, and predicting internal states of non‐human agents and objects could be partly dependent on low‐level processes that are distinct from those active when encountering human agents. A two‐stage process of anthropomorphism would suggest that these early low‐level perceptual processes are complemented at a later stage by using language derived from theory‐of‐mind. Anthropomorphism could be the end result of an otherwise largely perceptual process. This two‐stage process explains why measures that probe the ability to understand and predict the behaviour of the characters in the film were related to situational anthropomorphism. Inferring and understanding the behaviour of agents is a mixture of complex interactive predictive processes, ranging from perceptual processes (e.g., action observation and prediction; Cross et al., 2012) to theory‐of‐mind‐like processes. The extent to which interactions with non‐human agents trigger similar social cognitive mechanisms as do interactions with other humans is still up for debate. Thus, researchers have to entertain the possibility that interactions with non‐human agents might trigger processes that are not social in nature or do not match one‐to‐one with the processes that are active during interactions human counterparts (Cross & Ramsey, 2021; Henschel et al., 2020).

In order to better understand the way humans attribute socialness and even form social relationships with non‐human agents and objects, a better understanding of the role anthropomorphism and theory‐of‐mind play in these new interactions is warranted. Here, we used a multi‐dimensional, multi‐method, representative‐sample approach, combining both exploratory and confirmatory analyses, to provide new evidence on the relationship between these important facets of everyday social cognition. Future research combining brain and behavioural measures of anthropomorphism and theory‐of‐mind across similarly diverse samples will help us to better understand how people develop social bonds with humans and non‐human agents alike, across situations and individuals.

## AUTHOR CONTRIBUTIONS

**Ruud Hortensius** and **Michaela Kent**: Conceptualization. **Ruud Hortensius** and **Michaela Kent:** Data curation. **Ruud Hortensius** and **Michaela Kent**: Formal analysis. **Ruud Hortensius** and **Emily S. Cross**: Funding acquisition. **Ruud Hortensius**, **Michaela Kent**, **Laura Jastrzab**, and **Kohinoor Darda**: Investigation. **Ruud Hortensius** and **Michaela Kent**: Methodology. **Ruud Hortensius** and **Michaela Kent**: Project administration. **Ruud Hortensius**, **Michaela Kent**, **Kohinoor Darda**, **Laura Jastrzab**, **Kami Koldewyn**, **Richard Ramsey**, and **Emily S. Cross**: Resources. **Ruud Hortensius**, **Michaela Kent**, **Kohinoor Darda**, and **Richard Ramsey**: Software. **Ruud Hortensius** and **Emily S. Cross**: Supervision. **Kohinoor Darda** and **Richard Ramsey**: Validation. **Ruud Hortensius**, **Michaela Kent**, and **Kohinoor Darda**: Visualisation. **Ruud Hortensius** and **Michaela Kent**: Writing ‐ original draft preparation. **Ruud Hortensius**, **Michaela Kent**, **Kohinoor Darda**, **Laura Jastrzab**, **Kami Koldewyn**, **Richard Ramsey**, and **Emily S. Cross**: Writing ‐ review and editing.

## Supporting information

**Appendix S1**: Supporting Information

## Data Availability

Experiment 1: Data and code are publicly available on the OSF, https://osf.io/4nb2j/, and the whole‐brain group maps can be found at NeuroVault (K. J. Gorgolewski et al., 2015), https://neurovault.org/collections/6615/. Experiment 2: Data and code are publicly available on the OSF https://osf.io/a8pyr/.
