# Supplementary material for: Exploring the relationship between anthropomorphism and theory‐of‐mind in brain and behaviour
Source: Hum Brain Mapp. 2021 Jul 1;42(13):4224–41. doi: 10.1002/hbm.25542 (PMC8356980; doi:10.1002/hbm.25542)
Supplement: Supplementary file 1 — Appendix S1: Supporting Information [file HBM-42-4224-s001.docx]

Supplementary material for

**Exploring the relationship between anthropomorphism and Theory-of-Mind in brain and behaviour**

by *Ruud Hortensius^#^, Michaela Kent^#^, Kohinoor M. Darda, Laura Jastrzab, Kami Koldewyn, Richard Ramsey & Emily S. Cross*

**Experiment 1**

| **Table S1. Demographics per dataset** | | | |  |
| --- | --- | --- | --- | --- |
|  | Dataset 1 | Dataset 2 | Dataset 3 | Dataset 4 |
| Age | 20.07±1.94 | 25.43±4.72 | 25.73±5.35 | 21.95±3.05 |
| Women / Men | 20/9 | 22/13 | 12/10 | 0 / 22 |
| Mean ± standard deviations are reported for age. | | | | |

| **Table S2. Dispositional Anthropomorphism per dataset** | | | |  |
| --- | --- | --- | --- | --- |
|  | Dataset 1 | Dataset 2 | Dataset 3 | Dataset 4 |
| IDAQ | 48 (17) | 55 (16)* | 44.5 (29.5) | 47 (20.5) |
| IDAQ-NA | 88 (20) | 93 (13)* | 92 (16) | 93 (15.75) |
| Median and interquartile range (IQR) are reported. The Individual Differences in Anthropomorphism Questionnaire (Waytz, Cacioppo, et al., 2010) was used to measure dispositional anthropomorphism. IDAQ: dispositional anthropomorphism; IDAQ-NA: dispositional attribution in general. Scores can range from 0 – 150. Cronbach’s α = 0.80, 95% confidence interval [.75, .86] and 0.58 [.47, .70] for the IDAQ and IDAQ-NA respectively across datasets. *16 participants completed a version of the questionnaire with the scale ranging from 1-10 instead of 0-10. Excluding those participants resulted in a median (IQR) of 56 (21) and a Cronbach’s α of .80 [.74, .86] for the IDAQ, and a median (IQR) of 93 (13.5) and Cronbach’s α of .62 [.51, .73] for the IDAQ-NA. | | | | |

| **Table S3. BOLD acquisition parameters per dataset** | | | |  | |
| --- | --- | --- | --- | --- | --- |
|  | Dataset 1 | Dataset 2* | Dataset 3 | | Dataset 4 |
| Repetition time (ms) | 2000 | 2000 | 2000 | | 2000 |
| Echo time (ms) | 30 | 30 | 26 | | 13 / 31 |
| Voxel size (mm) | 3 x 3 x 3.5 | 3 isotropic | 2 isotropic | | 2.75 x 2.75 x 4 |
| Number of slices | 32 | 37 | 68 | | 32 |
| Flip angle | 83° | 77° | 60° | | 85° |
| Field of view (mm) | 240 x 240 | 210 x 210 | 192 x 192 | | 220 x 220 |
| Matrix size | 80 x 80 | 70 x 70 x 37 | 96 x 96 | | 80 x 80 |
| Slice acquisition | Ascending | Ascending | Ascending | | Ascending |
| Phase encoding | Anterior-posterior | Anterior-posterior | Anterior-posterior | | Anterior-posterior |
| Acquisition orientation | Co-planar with AC–PC | Co-planar with AC–PC | Co-planar with AC–PC | | 45° angle from AC-PC |
| Number of volumes | 180 | 175 | 175 | | 174 |
| Pulse sequence | EPI | EPI | multi-band EPI (factor = 2) | | multi-echo EPI |
| Parallel imaging parameters | SENSE, P reduction: 2 | GRAPPA, acceleration factor (2) | GRAPPA, acceleration factor (2) | | GRAPPA acceleration factor (3) |
| 3T MRI system | Philips Achieva | Siemens TrioTim | Siemens TrioTim | | Siemens TrioTim |
| Site | Bangor University | University of Glasgow | University of Glasgow | | University of Glasgow |
| EPI: echo planar image. *For the first 2 participants, the following parameters were different: voxel size 3.121 x 3.121 x 3.410 mm, number of slices: 38, flip angle: 90°, field of view: 206 x 206, matrix size: 66 x 66, number of volumes: 170. Note, these participants are not included in the whole-brain second-level analysis, but are included in the ROI analysis. | | | | | |

| **Table S4. Functional image-quality metrics per dataset** | | | | |  |
| --- | --- | --- | --- | --- | --- |
|  | Dataset 1 | Dataset 2 | Dataset 3 | Dataset 4 |  |
| SNR | 4.831 ± 0.288 | 5.319 ± 0.330 | 4.946 ± 0.272 | 5.540 ± 0.862 |  |
| tSNR | 75.726 ± 16.990 | 42.628 ± 5.805 | 37.326 ± 3.469 | 47.830 ± 14.122 |  |
| EFC | 0.412 ± 0.024 | 0.541 ± 0.024 | 0.543 ± 0.027 | 0.543 ± 0.036 |  |
| FBER | 277129 ± 58574 | 1503 ± 249 | 10609 ± 3752 | 3125 ± 1400 |  |
| GSR_x | -0.005 ± 0.004 | 0.003 ± 0.007 | -0.007 ± 0.006 | -0.005 ± 0.009 |  |
| GSR_y | 0.011 ± 0.004 | 0.025 ± 0.008 | 0.017 ± 0.009 | 0.032 ± 0.020 |  |
| DVARS_nstd | 18.383 ± 3.249 | 31.950 ± 3.904 | 43.564 ± 3.239 | 32.676 ± 13.408 |  |
| DVARS_std | 1.197 ± 0.123 | 1.094 ± 0.048 | 1.175 ± 0.059 | 1.100 ± 0.039 |  |
| DVARS_vstd | 0.994 ± 0.075 | 1.002 ± 0.031 | 1.008 ± 0.013 | 1.011 ± 0.019 |  |
| GCOR | 0.031 ± 0.016 | 0.009 ± 0.006 | 0.005 ± 0.002 | 0.008 ± 0.004 |  |
| FD_mean | 0.126 ± 0.050 | 0.112 ± 0.053 | 0.093 ± 0.048 | 0.163 ± 0.063 |  |
| FD_number | 19.821 ± 19.501 | 15.800 ± 26.376 | 14.048 ± 19.351 | 36.477 ± 28.406 |  |
| FD_percentage | 11.012 ± 10.834 | 9.039 ± 15.079 | 8.034 ± 11.068 | 20.997 ± 16.340 |  |
| Spikes | 2.500 ± 7.555 | 0.371 ± 0.973 | 0.381 ± 0.921 | 0.977 ± 2.367 |  |
| AOR | 0.004 ± 0.005 | 0.002 ± 0.002 | 0.001 ± 0.001 | 0.002 ± 0.001 |  |
| AQI | 0.006 ± 0.004 | 0.015 ± 0.006 | 0.017 ± 0.003 | 0.014 ± 0.012 |  |
| Mean ± standard deviations are reported for the image-quality metrics derived from MRIQC (version 0.14.2) (Esteban et al., 2017) for each dataset. The impact of noise is assessed by the signal-to-noise ratio (SNR) (grey / outside) and temporal signal-to-noise ratio (tSNR), spatial distribution of information is assessed by the entropy-focus criterion (EFC; indicating ghosting and blurring due to head-motion), foreground-background energy ratio (FBER; inside vs. outside voxels), and ghost to signal ratio (GSR; mean signal in area susceptible to ghosting along phase-encoding axes: x and y), while artifacts are indicated by DVARS (average change in mean intensity between each functional volume in the run; normalised and voxel-wise standardised are reported as well ), global correlation (GCOR; average correlation across all pairs of voxel time series inside the brain), mean and number of framewise displacement (FD; mean, number and percentage of timepoints with head-motion across X, Y, Z directions and rotations > .20mm), and spikes (indicating high-frequency movements). MRIQC also calculates AFNI’s outlier ratio (AOR; number of outliers / total number of timepoints) and quality index (AQI; assessing the run deviation for the subject’s norm). Please note that these image-quality metrics are no-reference. | | | | | |

| **Table S5. Coordinates for the Theory-of-Mind regions** | | | |
| --- | --- | --- | --- |
| *Region* | *MNI Coordinates* | | |
|  | *x* | *y* | *z* |
| Dorsomedial prefrontal cortex | -6 | 54 | 36 |
| Middle medial prefrontal cortex | -4 | 58 | 16 |
| Ventromedial prefrontal cortex | -4 | 56 | -16 |
| Precuneus | 0 | -54 | 34 |
| Left temporoparietal junction | -48 | -62 | 30 |
| Right temporoparietal junction | 48 | -60 | 30 |
| Coordinates are based on Richardson et al. (2018). A 9mm sphere is used for each region. | | | |

| **Table S6. Coordinates for the Pain Matrix regions** | | | |
| --- | --- | --- | --- |
| *Parcel* | *MNI Coordinates* | | |
|  | *x* | *y* | *z* |
| Anterior middle cingulate cortex | 0 | 2 | 42 |
| Left secondary somatosensory cortex | -62 | -32 | 34 |
| Right secondary somatosensory cortex | 60 | -28 | 38 |
| Left insula | -42 | -2 | -4 |
| Right insula | 42 | 6 | -6 |
| Left middle frontal gyrus | -46 | 36 | 14 |
| Right middle frontal gyrus | 50 | 42 | 12 |
| Coordinates are based on Richardson et al. (2018). A 9mm sphere is used for each region. | | | |


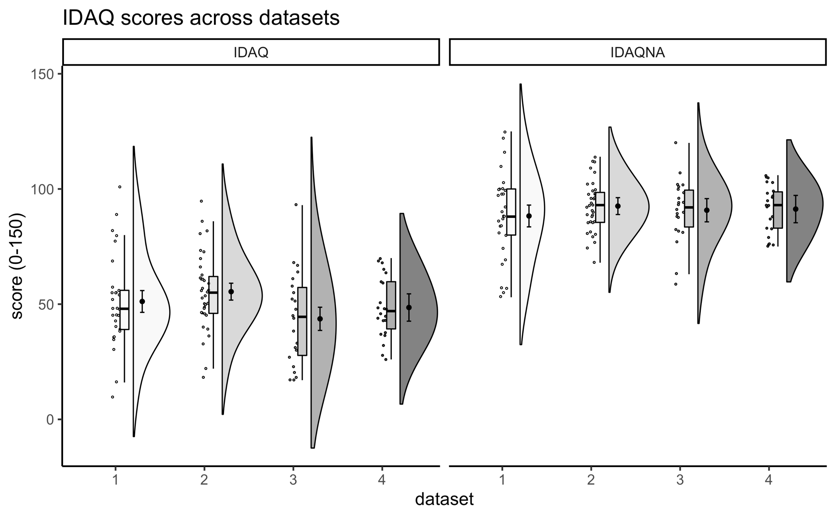


**Figure S1. Individual Differences in Anthropomorphism Questionnaire scores across datasets and scale.** The scores for the anthropomorphic scale (IDAQ) and nonanthropomorphic (IDAQ-NA) are shown using rain clouds plots with errors bars reflecting 95% confidence intervals (Allen et al., 2019).


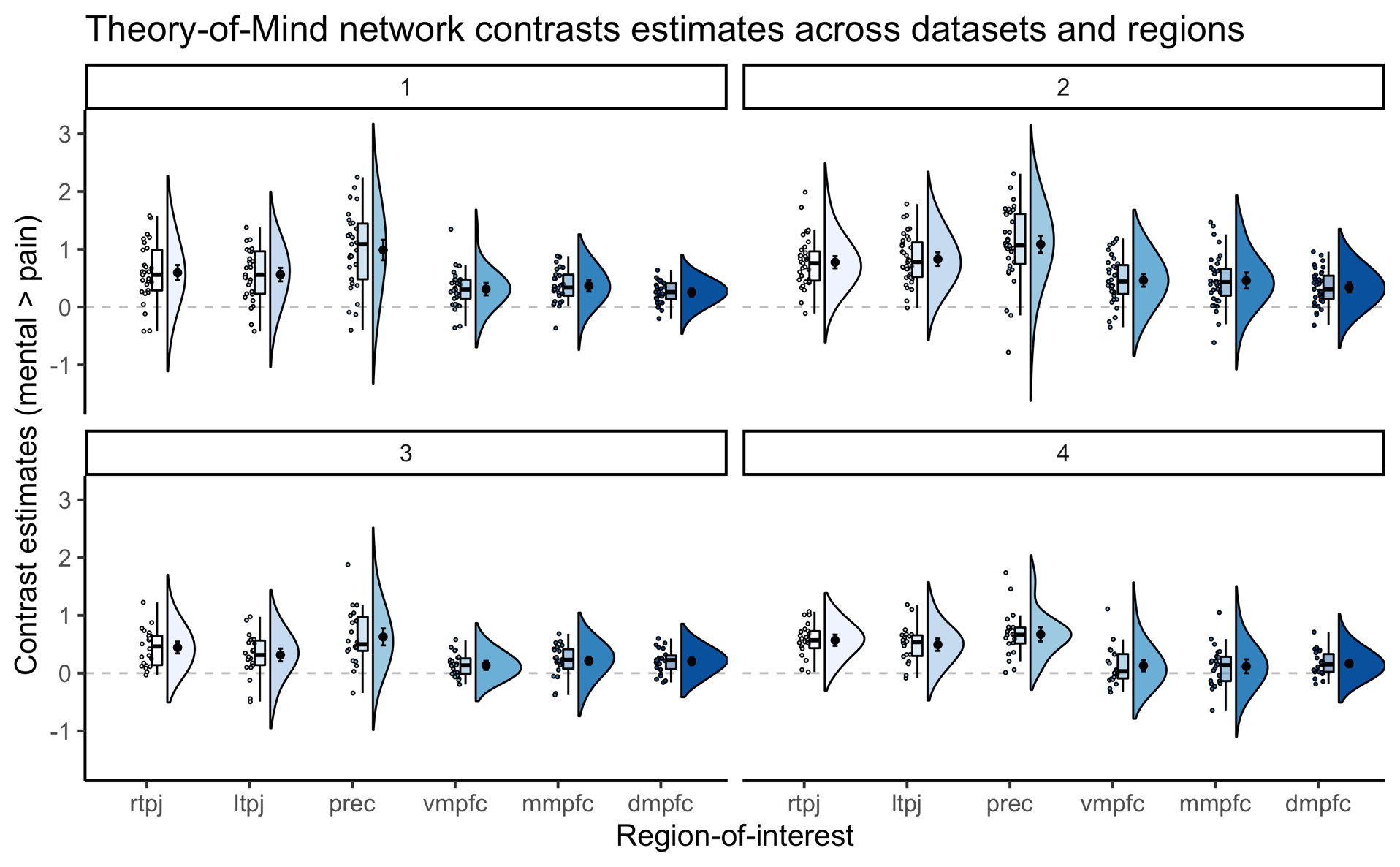


**Figure S2. Activation in regions of the Theory-of-Mind network for each of the datasets.** Contrast values for the mental > pain contrast are shown for the six regions of the Theory-of-Mind network. Dashed line indicates a contrast value of zero and errors bars reflecting 95% confidence intervals, dmpfc, dorsomedial prefrontal cortex; mmpfc, middle medial prefrontal cortex; prec, precuneus; rtpj and ltpj, right and left temporoparietal junction; vmpfc, ventromedial prefrontal cortex.


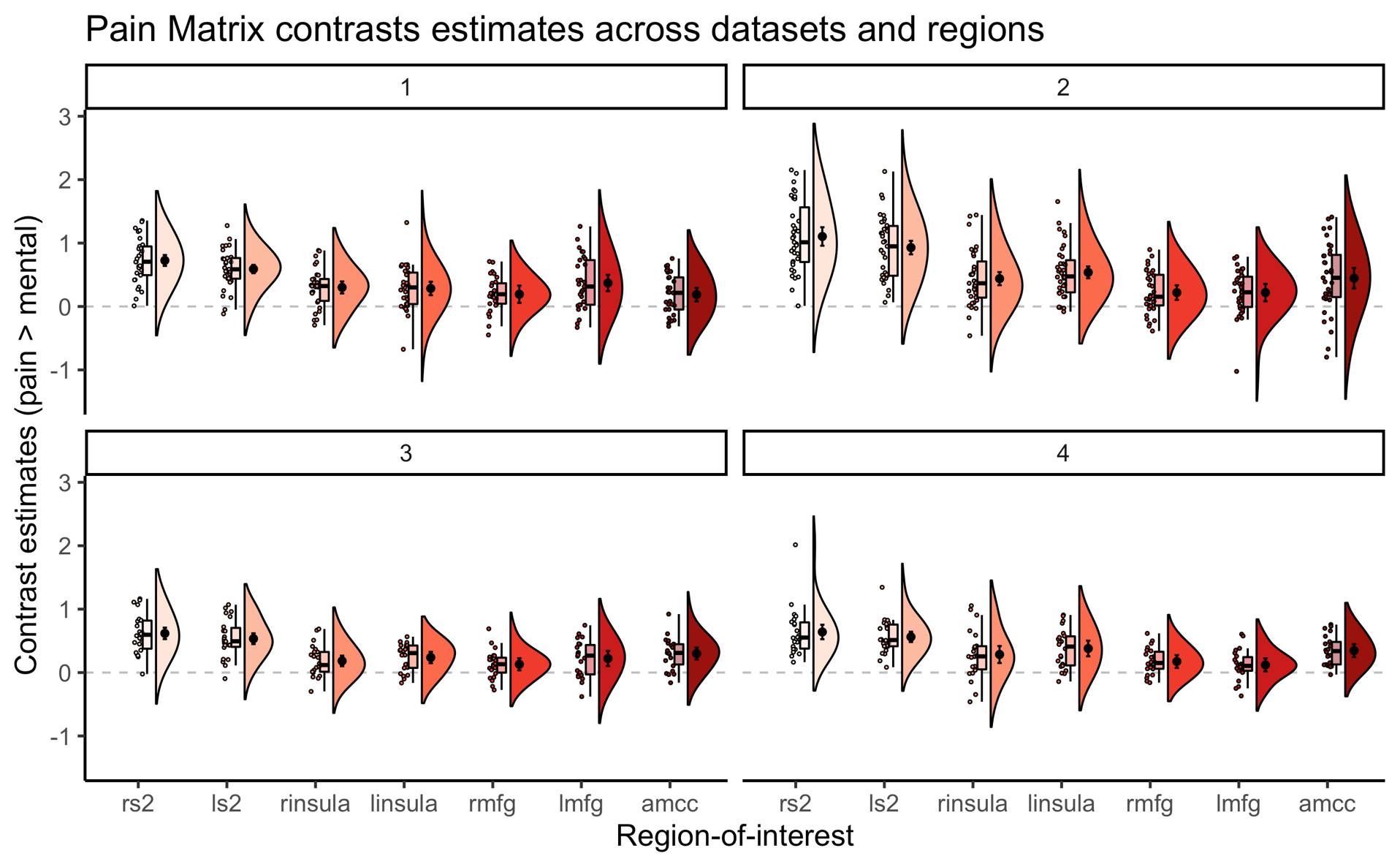


**Figure S3. Activation in regions of the Pain Matrix for each of the datasets.** Contrast values for pain > mental are shown for the seven regions of the Pain Matrix. Dashed line indicates a contrast value of zero and errors bars reflecting 95% confidence intervals, rs2 and ls2, bilateral secondary somatosensory cortex; rinsula and linsula, bilateral insula; rmfg and lmfg, bilateral middle frontal gyrus; amcc, anterior middle cingulate cortex.

**Figure S4. Whole-brain analyses showed robust activation across the Theory-of-Mind network and the Pain Matrix.** Contrast maps for mental > pain (in yellow) and pain > mental (in blue) are shown for each dataset (*p* <0.001 uncorrected, k=10, with an average grey matter mask applied). Numbers 1-6 indicate regions of the Theory-of-Mind network: bilateral temporoparietal junction (1 and 2), precuneus (3), and dorsomedial (4), middle medial (5), and ventromedial prefrontal cortex (6). Number 7-11 indicate regions of the Pain Matrix: bilateral second somatosensory cortex (7 and 10), bilateral middle frontal gyrus (8 and 9) and anterior middle cingulate cortex (11), with the bilateral insula not shown. For dataset 2, the first two participants are not included in the whole-brain analyses due to partially different scan parameters. For dataset 4, activation in the prefrontal regions of the Theory-of-Mind network did not survive this threshold. All group maps can be found on NeuroVault: <https://neurovault.org/collections/6615/>.

**
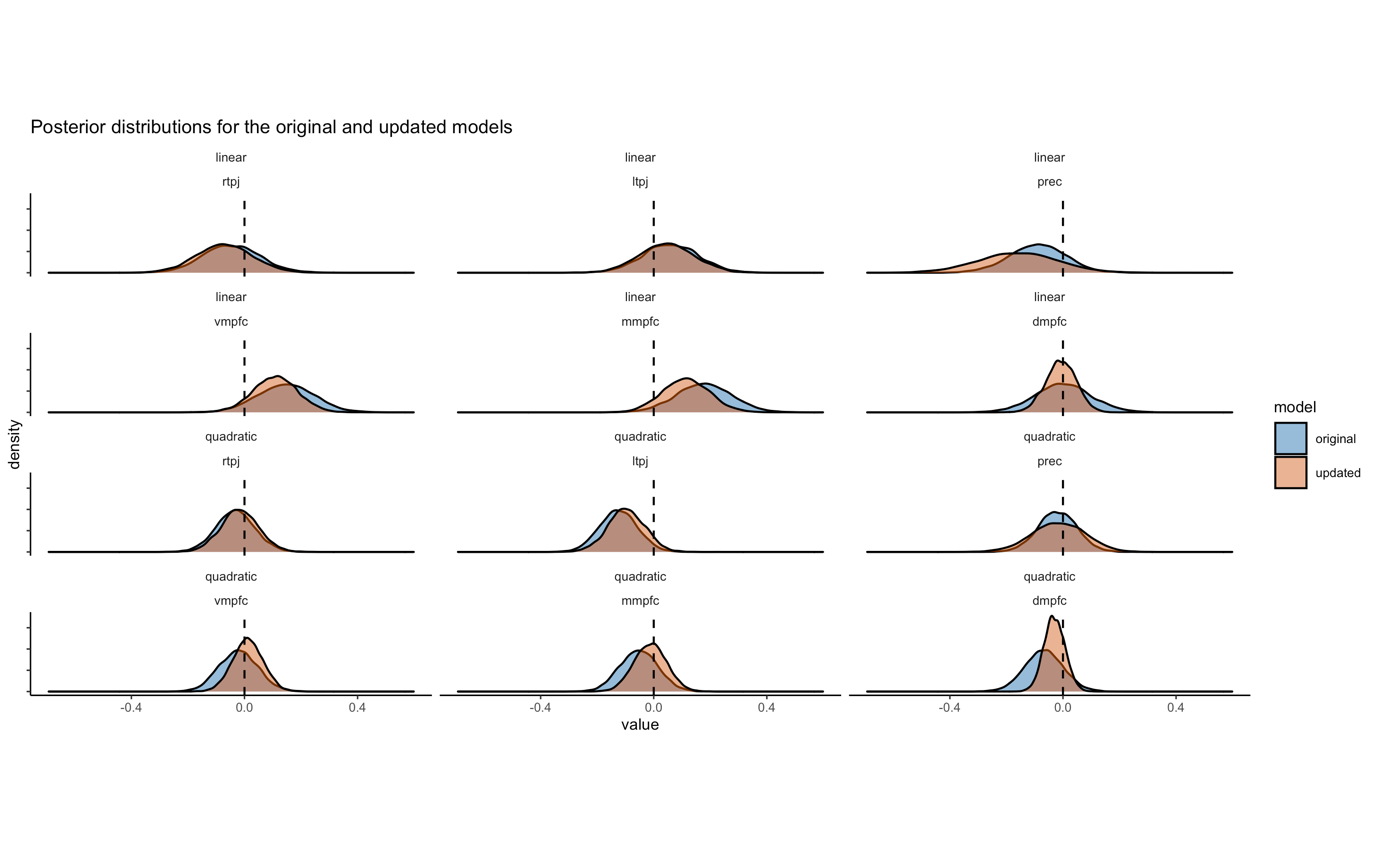
**

**Figure S5. Posterior distribution for the original and updated Theory-of-Mind models.** We re-ran the main analysis excluding the 16 participants that completed a different version of the IDAQ version. The posterior distributions of these models overlap with the distributions of the original model with the complete sample. dmpfc, dorsomedial prefrontal cortex; mmpfc, middle medial prefrontal cortex; prec, precuneus; rtpj and ltpj, right and left temporoparietal junction; vmpfc, ventromedial prefrontal cortex.

**
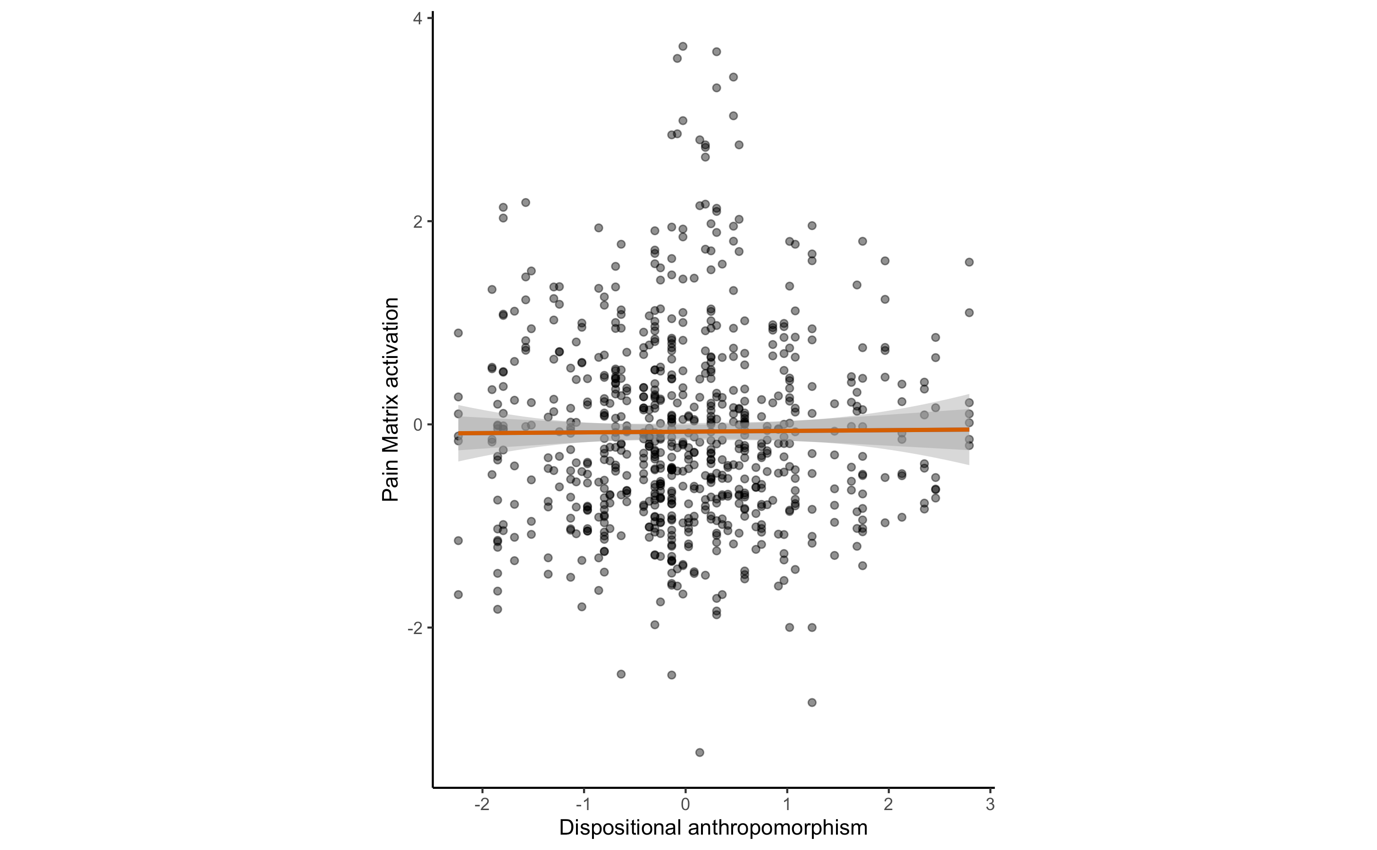
**

**Figure S6. Dispositional anthropomorphism and overall Pain Matrix activation.** Neither a linear (blue line) or quadratic relationship (red line) was visible between dispositional anthropomorphism and activity across the Pain Matrix during the observation of scenes that trigger pain perception compared to scenes that trigger mentalising. Indices are centered and scaled.

**
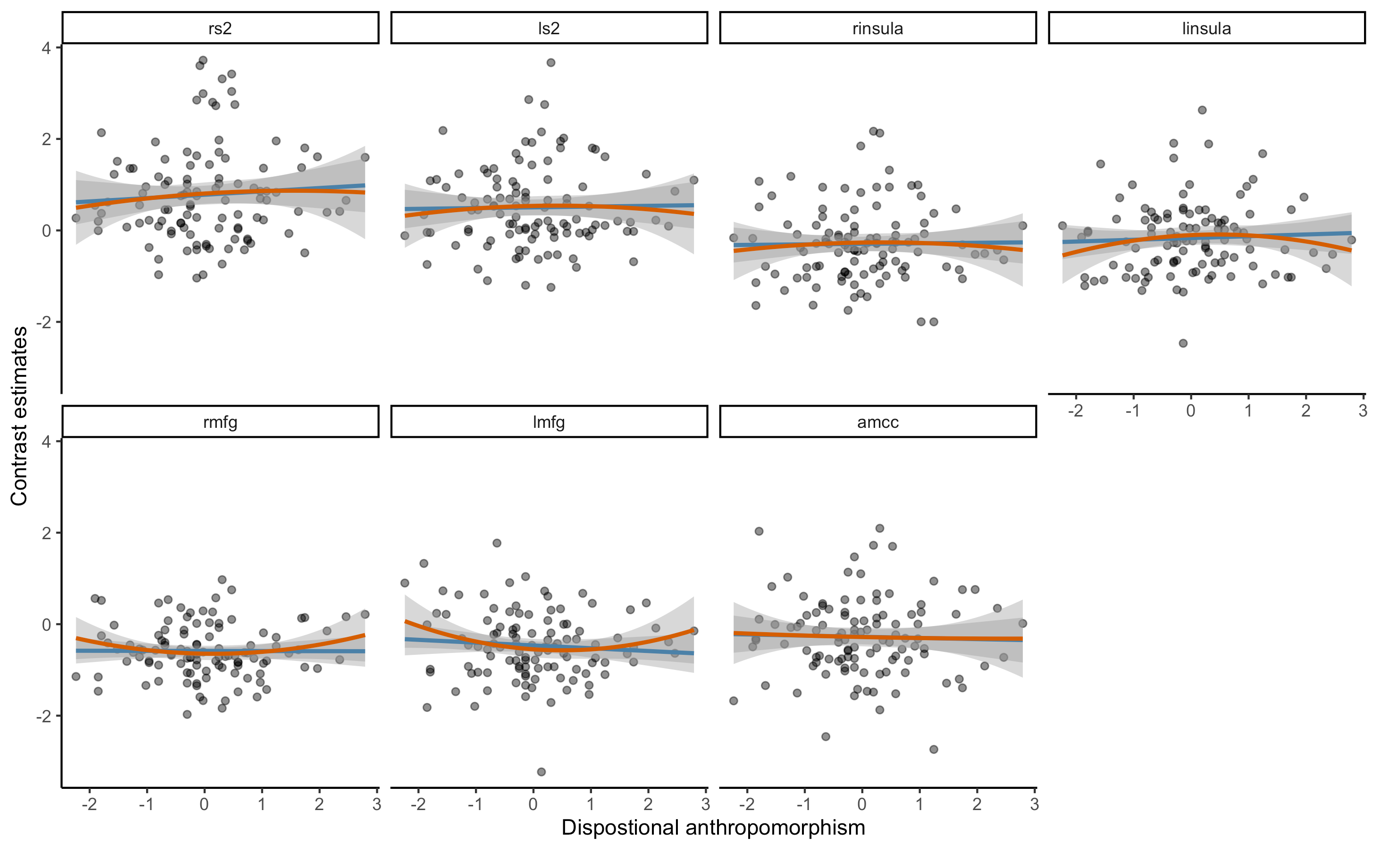
**

**Figure S7. Dispositional anthropomorphism and activity within the regions of the Pain Matrix.** No linear (blue line) or quadratic relationship (red line) was visible between dispositional anthropomorphism and activity across the Pain Matrix during the observation of scenes that trigger pain perception compared to scenes that trigger mentalising. Indices are centered and scaled, rs2 and ls2: right and left second somatosensory cortex, rinsula and linsula: right and left insula, rmfg and lmfg: right and left middle frontal gyrus, amcc: anterior middle cingulate cortex.

**
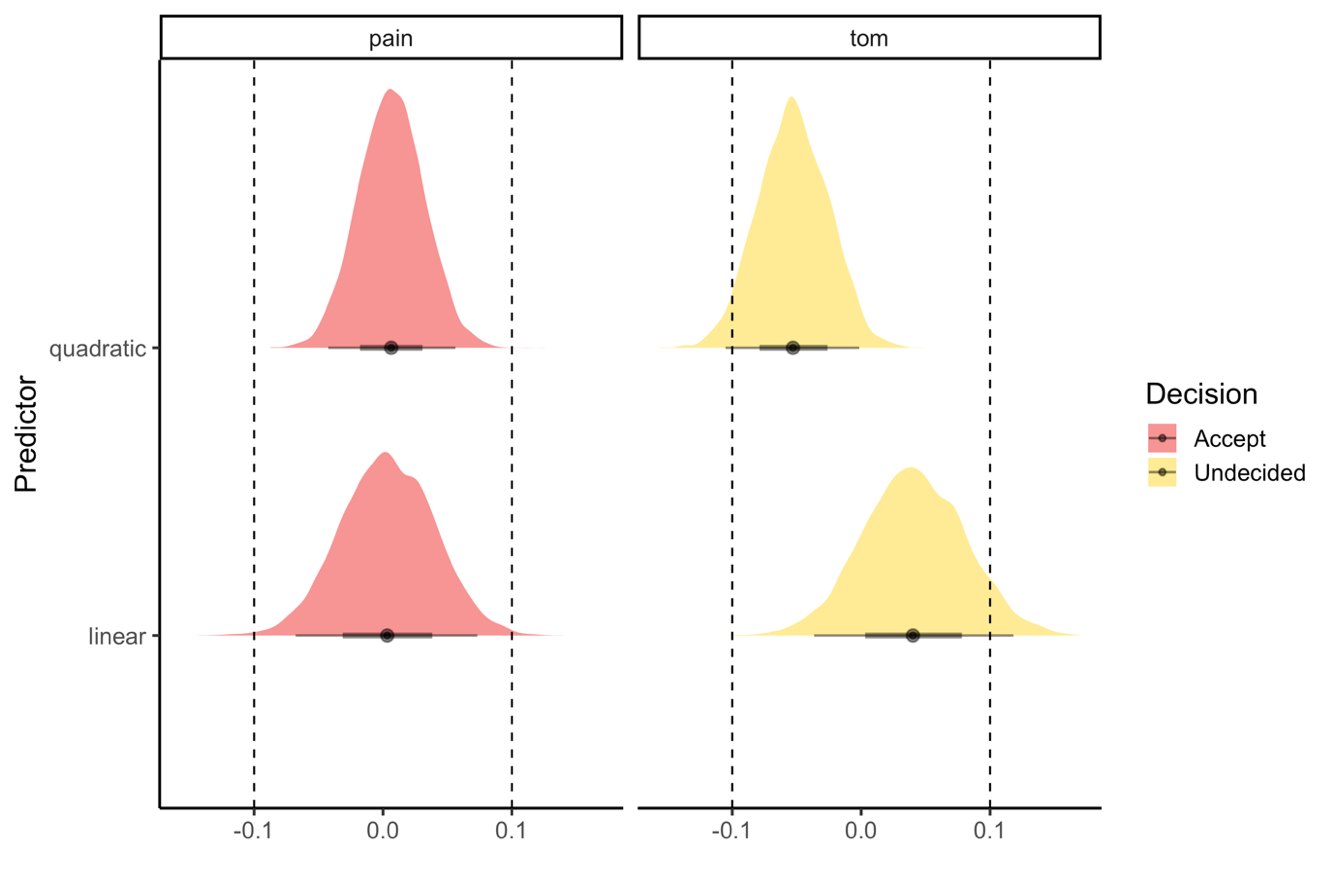
**

**Figure S8. Posterior distribution for the linear and quadratic dispositional anthropomorphism predictor for activity across the Theory-of-Mind network and Pain Matrix.** While based on the highest density interval of the posterior distribution and a region of practical equivalence around the null decision rule the null can be accepted for the Pain Matrix, there is inconclusive evidence for the Theory-of-Mind network.

**
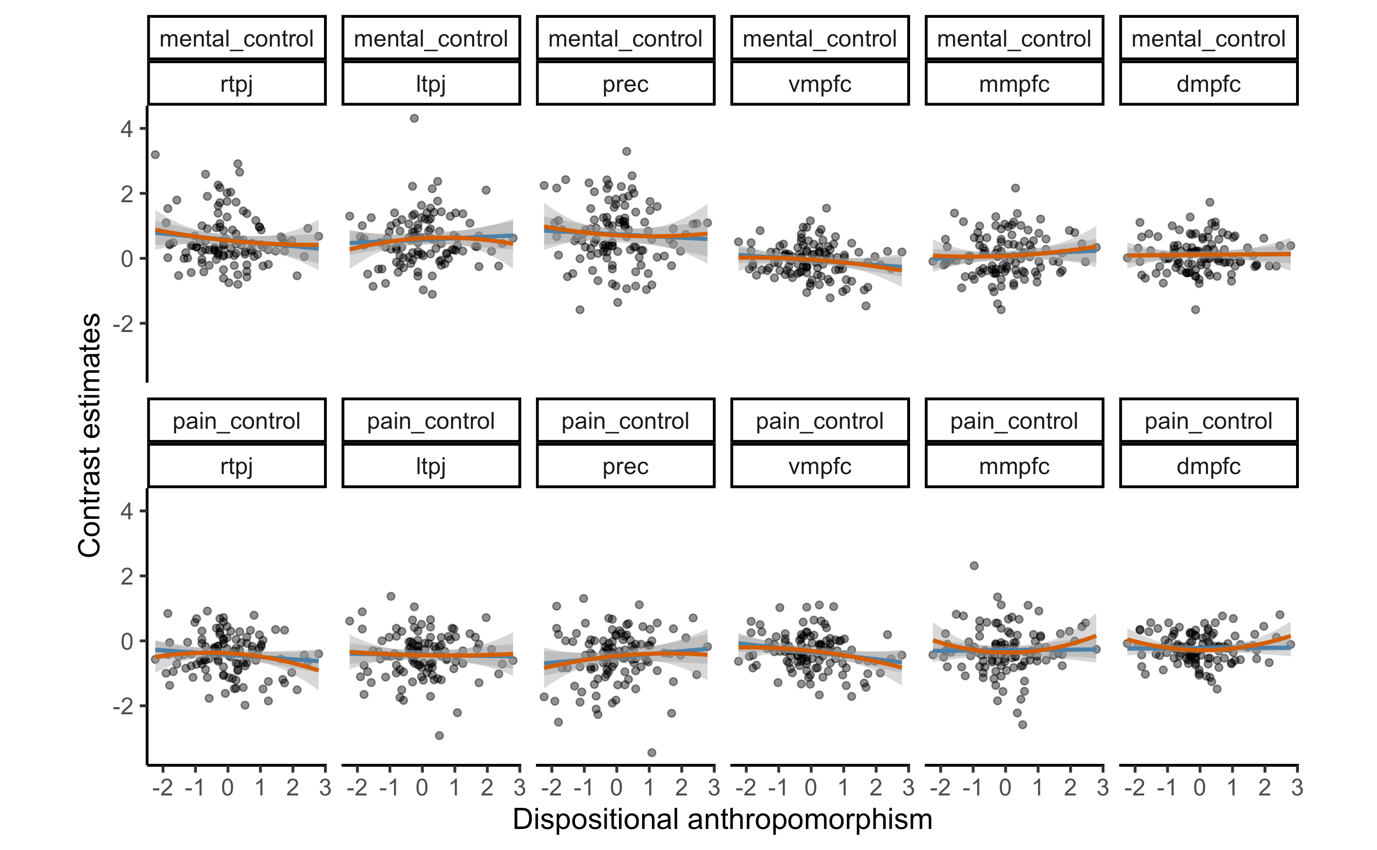
**

**Figure S9. Dispositional anthropomorphism and activity within the regions of the Theory-of-Mind network across contrasts.** No linear (blue line) or quadratic relationship (red line) was visible between dispositional anthropomorphism and activity across the Theory-of-Mind during the observation of scenes that trigger mentalising compared to scenes not related to the main characters (mental_control) or scenes that trigger pain perception compared to scenes not related to the main characters (pain_control). Indices are centered and scaled, hand-coded events from Jacoby and colleagues (2016) were used, dmpfc, dorsomedial prefrontal cortex; mmpfc, middle medial prefrontal cortex; prec, precuneus; rtpj and ltpj, right and left temporoparietal junction; vmpfc, ventromedial prefrontal cortex.


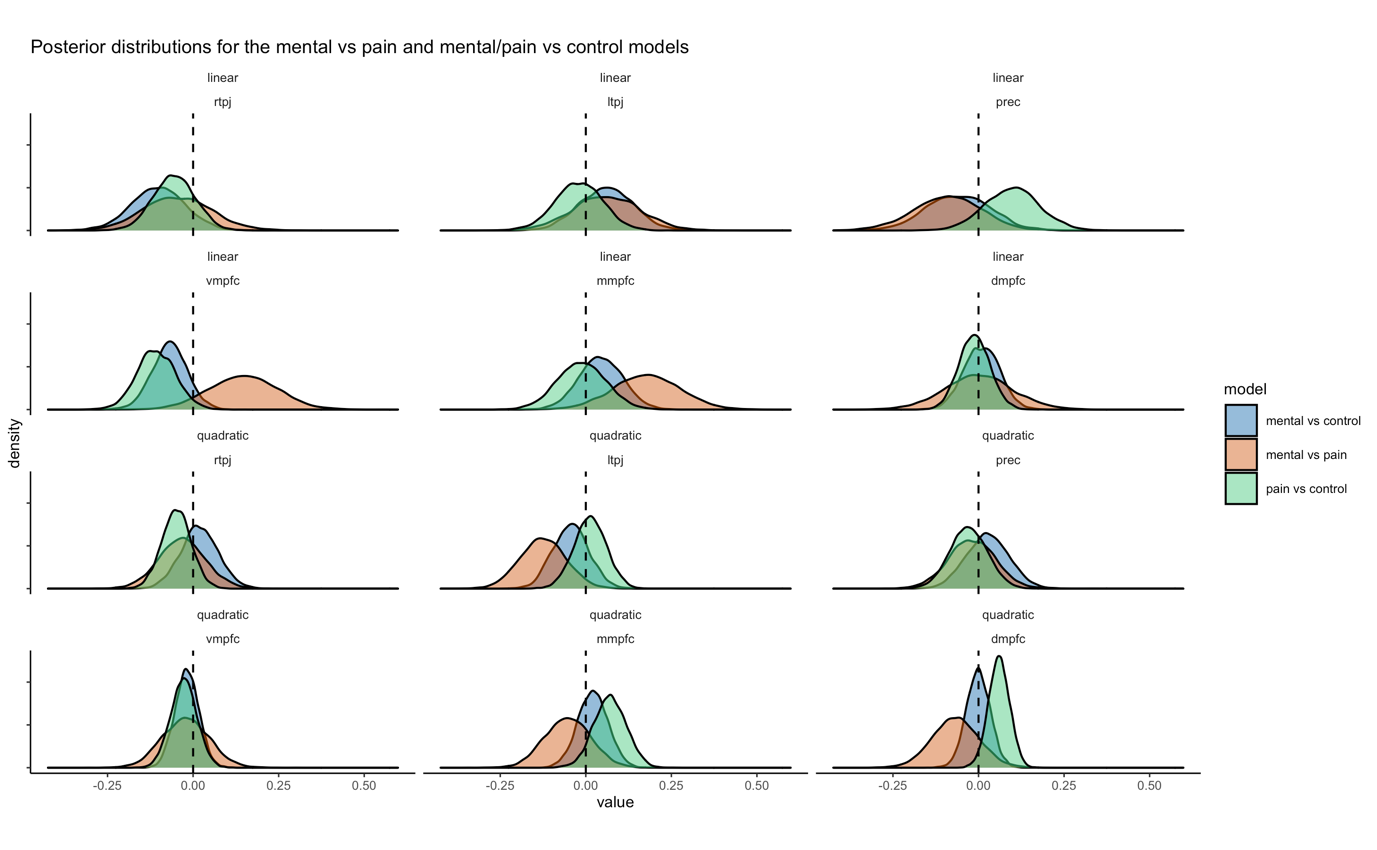
**Figure S10. Posterior distribution for the model for each Theory-of-Mind contrast.** Similar results are obtained when running the main analyses with the original contrast, comparing scenes that trigger mentalising compared to scenes that trigger pain perception (mental vs pain), as when running control models comparing scenes that trigger mentalising or pain perception with scenes that not related to the main characters (mental vs control or pain vs control). dmpfc, dorsomedial prefrontal cortex; mmpfc, middle medial prefrontal cortex; prec, precuneus; rtpj and ltpj, right and left temporoparietal junction; vmpfc, ventromedial prefrontal cortex.

| **Table S7. Estimated posterior regression coefficient for the linear and quadratic anthropomorphism predictor across each Pain Matrix region** | | | | | | | | | | | | | | |
| --- | --- | --- | --- | --- | --- | --- | --- | --- | --- | --- | --- | --- | --- | --- |
|  | **rs2** | | **ls2** | | **rinsula** | | **linsula** | | **rmfg** | | **lmfg** | | **amcc** | |
| *Predictors* | *Estimates* | *CI (95%)* | *Estimates* | *CI (95%)* | *Estimates* | *CI (95%)* | *Estimates* | *CI (95%)* | *Estimates* | *CI (95%)* | *Estimates* | *CI (95%)* | *Estimates* | *CI (95%)* |
| Intercept | 0.03 | -0.21 – 0.26 | 0.04 | -0.20 – 0.26 | 0.04 | -0.20 – 0.27 | 0.08 | -0.16 – 0.31 | -0.10 | -0.34 – 0.15 | -0.11 | -0.35 – 0.12 | -0.00 | -0.24 – 0.23 |
| linear predictor | 0.08 | -0.12 – 0.27 | 0.03 | -0.17 – 0.23 | 0.02 | -0.17 – 0.22 | 0.07 | -0.12 – 0.27 | -0.03 | -0.22 – 0.16 | -0.11 | -0.31 – 0.08 | -0.03 | -0.22 – 0.18 |
| quadratic predictor | -0.02 | -0.16 – 0.11 | -0.04 | -0.17 – 0.09 | -0.04 | -0.17 – 0.10 | -0.08 | -0.21 – 0.05 | 0.10 | -0.04 – 0.23 | 0.11 | -0.02 – 0.24 | 0.01 | -0.13 – 0.14 |
| R^2^ Bayes | 0.018 | | 0.015 | | 0.015 | | 0.026 | | 0.031 | | 0.042 | | 0.014 | |
| CI (95): 95% credibility interval, rs2 and ls2: right and left second somatosensory cortex, rinsula and linsula: right and left insula, rmfg and lmfg: right and left middle frontal gyrus, amcc: anterior middle cingulate cortex. | | | | | | | | | | | | | | |

| **Table S8. Estimated posterior regression coefficient for the linear and quadratic anthropomorphism predictor within the regions of the Theory-of-Mind network across contrasts.** | | | | | | | | |
| --- | --- | --- | --- | --- | --- | --- | --- | --- |
|  | **mental events** | | **pain events** | | **social events** | | **all events** | |
| *Predictors* | *Estimates* | *CI (95%)* | *Estimates* | *CI (95%)* | *Estimates* | *CI (95%)* | *Estimates* | *CI (95%)* |
| Intercept | 0.34 | 0.26 – 0.41 | -0.37 | -0.44 – -0.31 | -0.05 | -0.11 – -0.01 | 0.08 | -0.07 – 0.22 |
| linear predictor | -0.02 | -0.08 – 0.04 | -0.02 | -0.07 – 0.04 | -0.05 | -0.10 – -0.01 | -0.09 | -0.22 – 0.03 |
| quadratic predictor | 0.00 | -0.04 – 0.04 | 0.01 | -0.03 – 0.04 | -0.00 | -0.03 – 0.03 | 0.01 | -0.08 – 0.09 |
| R^2^ Bayes | 0.003 | | 0.003 | | 0.011 | | 0.005 | |
| CI (95): 95% credibility interval, hand-coded events from Jacoby and colleagues (2016) were used. Mental events trigger mentalising, pain events trigger pain perception, social events are events containing interactions between the characters without triggering internal state prediction. All events are compared to events not related to the main characters individually and combined (all events). | | | | | | | | |

| **Table S9. Estimated posterior regression coefficient for the task-independent region-of-interest** | | | | |
| --- | --- | --- | --- | --- |
|  | **ltpj** | | **mpfc-acc** | |
| *Predictors* | *Estimates* | *CI (95%)* | *Estimates* | *CI (95%)* |
| Intercept | 0.30 | 0.12 – 0.49 | -0.22 | -0.48 – 0.04 |
| linear predictor | 0.06 | -0.10 – 0.21 | 0.08 | -0.15 – 0.30 |
| quadratic predictor | -0.08 | -0.19 – 0.03 | -0.01 | -0.15 – 0.15 |
| R^2^ Bayes | 0.032 | | 0.017 | |
| CI (95): 95% credibility interval, MNI coordinates for the left temporoparietal junction (TPJ) are from Cullen et al. (2014) [-45, -54, 27], while the coordinates for the cluster overlapping with the medial prefrontal and- anterior cingulate cortex (mPFC-ACC) are from Waytz et al. (2010) [4, 44, -10]. The coordinates from Waytz et al. (2010) were transformed from Taliarach to MNI using the tal2icbm_spm function (Lancaster et al., 2007). | | | | |

**Experiment 2**


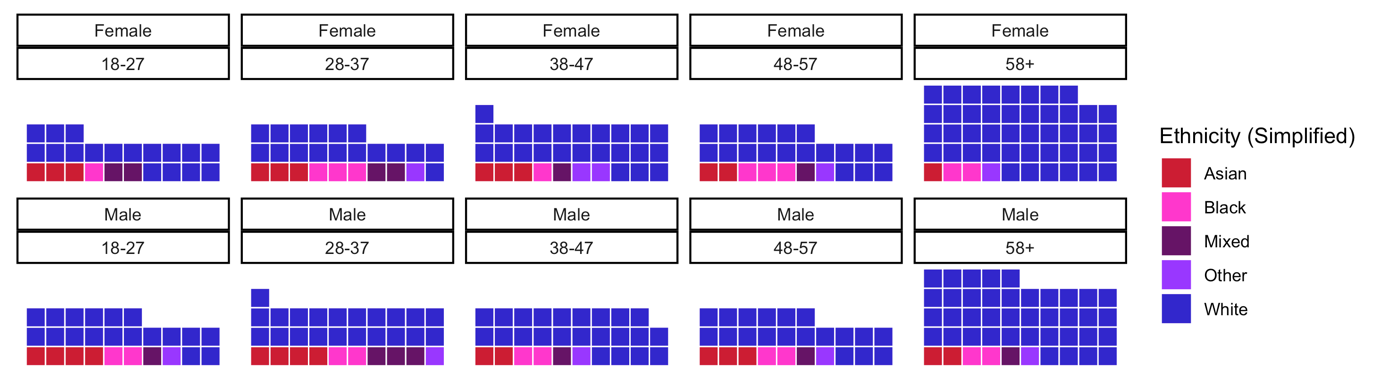


**Figure S11. Demographics for the representative sample stratified across age, sex, and ethnicity recruited for Experiment 2.** Ethnicity (simplified) is displayed across age bins for female and male participants. One square is one participant (*n* = 311).

**Supplemental Results – Experiment 2**

*The relationship between behavioural indices and neural correlates on the Theory-of-Mind False Belief Task.*

**Method**

*Participants.* In order to test the relationship between behavioural and neural correlates of the False Belief task, we use data from 50 participants from previously published work (Darda et al., 2018) and data from 36 participants that was previously collected for an experiment unrelated to the current work (Darda & Ramsey, in prep). Thus, data from eighty-six (Mean_age_=23.24, SD_age_=4.24; 46 females) participants on the Theory-of-Mind Network Localiser (False Belief) task was used in the current study. Participants gave informed consent in line with the guidelines set by the Research Ethics and Governance Committee of the School of Psychology at Bangor University (2016-15863), were right-handed, had normal or corrected-to-normal vision, and reported no history of neurological damage.

*Design and Procedure.* Along with the False Belief task, all participants also did other tasks unrelated to the current study (as described in Experiment 2 in Darda et al., 2018). Thirty-six participants additionally also completed a flanker task (Eriksen & Eriksen, 1974), a mirror neuron network localiser task (Spunt & Lieberman, 2012) and a body perception network localiser task (Downing et al., 2007).

*Theory-of-Mind network localiser - the false belief task.* To localize brain regions involved in mental state reasoning, we used a paradigm developed by Dodell-Feder and colleagues (2011; saxelab.mit.edu/superloc.php). This localizer task includes 20 stories, each describing a false representation. Ten stories included out-of-date beliefs (the false belief condition), and the other 10 included out-of-date physical representations (photographs/maps; the false photograph condition). The false belief > false photograph contrast has been shown in prior work to robustly activate regions involved in mentalizing (Dufour et al., 2013). All trials consisted of a story (10 sec), followed by a true or false question (4 sec). Each story was separated by a 12-sec rest period. The order of the stories and conditions was the same for all participants. Each participant completed two runs of this task, with five trials per condition presented in each run.

**Behavioural Data Acquisition**

Accuracy and reaction time (RT) were recorded on the False Belief task for all 20 trials. RT was measured as the time from presentation of the true/false question to when participants made a response. Accuracy was calculated as number of correct trials divided by total number of trials. For the current analyses, we were interested in finding whether behavioural data (accuracy and RT) when mentalising or understanding others’ mental states was related to responses in the Theory-of-Mind network in the brain. Therefore, for all further analyses, we use accuracy and RT information recorded for the false belief condition (and not the false photograph condition).

**fMRI Data Acquisition**

For detailed fMRI data acquisition, preprocessing, and functional region-of-interest (fROI) analysis parameters, please refer to Darda et al. (2018). 136 volumes were collected on each run of the Theory-of-Mind network localiser task. The design matrix consisted of regressors for each experimental condition (“Belief” and “Photo” for the Theory-of-Mind localizer). The onset and duration of each condition was specified and convolved with the standard hemodynamic response function. Contrast images were then calculated for each individual subject to identify regions that responded to mentalizing (belief > photo). For the localiser task analysis, an across-run cross-validation approach was used (Nieto-Castañón & Fedorenko, 2012) to ensure that data used for defining fROIs were independent of data used for estimating response (Kriegeskorte et al., 2009).

For the Theory-of-Mind network, six parcels were derived in the same way as in the main analyses of the current paper (using MNI coordinates from Richardson et al., 2018). These parcels included ventromedial, middle medial, and dorsomedial prefrontal cortex (vMPFC, mMPFC, dMPFC), bilateral temporoparietal junction (lTPJ, rTPJ) and the precuneus and were combined into a number-labelled parcellated image using Marsbar. For each individual, this image or mask was used to constrain the selection of subject-specific fROIs. The belief > photo contrast was used, and the top 10% of voxels (based on *t* values) within each parcel were defined as that individual's fROI. Percent signal change responses to the localizer contrast Belief > Photo were estimated and investigated using a one-sampled t-test. False discovery rate (FDR) multiple-comparison correction (p < .05) was used to correct for the number of fROIs in each functional network.

**Behavioural and fMRI Data Analysis**

Percent signal change values in the six ROIs as well as accuracy and RT data on the false belief condition were used in the current analyses. In order to investigate the relationship between behavioural indices on the false belief task and responses in the Theory-of-Mind network, for each ROI individually, a Pearson’s correlation was performed on both accuracy and RT data separately.

**Results**

**Table S8** shows the results from the correlation analyses for each ROI. Results showed a significant positive correlation between accuracy and average Theory-of-Mind network response, and a significant negative correlation between RT and average Theory-of-Mind network response. Thus, participants who were more accurate on the task and responded faster showed a higher response on average in the Theory-of-Mind network (**Figure S10**). All ROIs showed a significant positive correlation with accuracy. All ROIs except mMPFC and dMPFC showed a significant negative correlation with RT (**Table S8**). Both mMPFC and dMPFC showed a negative relationship between RT and ROI response but this did not pass our statistical thresholding.

| **Table S10. Pearson r and *p*-values for the correlation analyses for each region-of-interest, and for reaction times and accuracy separately.** | | | | |
| --- | --- | --- | --- | --- |
| Region | Indices | Pearson’s r | *p*-value | Significance |
| Theory-of-Mind network averaged | Acc | 0.43 | <0.001 | ** |
|  | RT | -0.3 | 0.006 | * |
| Dorsomedial prefrontal cortex | Acc | 0.25 | 0.02 | * |
|  | RT | -0.16 | 0.15 |  |
| Middle medial prefrontal cortex | Acc | 0.28 | 0.008 | * |
|  | RT | -0.12 | 0.28 |  |
| Ventromedial prefrontal cortex | Acc | 0.33 | 0.002 | * |
|  | RT | -0.22 | 0.042 | * |
| Precuneus | Acc | 0.46 | <.001 | ** |
|  | RT | -0.37 | 0.001 | ** |
| Left temporoparietal junction | Acc | 0.29 | 0.007 | * |
|  | RT | -0.21 | 0.049 | * |
| Right temporoparietal junction | Acc | 0.26 | 0.014 | * |
|  | RT | -0.22 | 0.044 | * |
| *=significant at p<.05, **=significant at p<.005, Acc = accuracy, RT=reaction time, NHST = Null Hypothesis Significance Testing. | | | | |


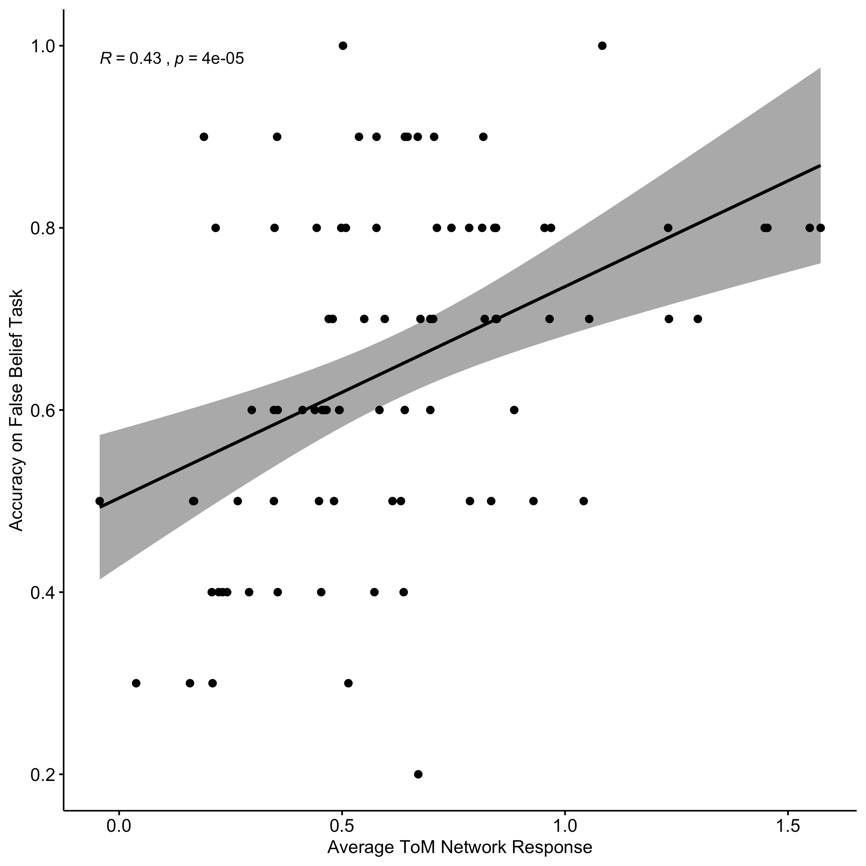

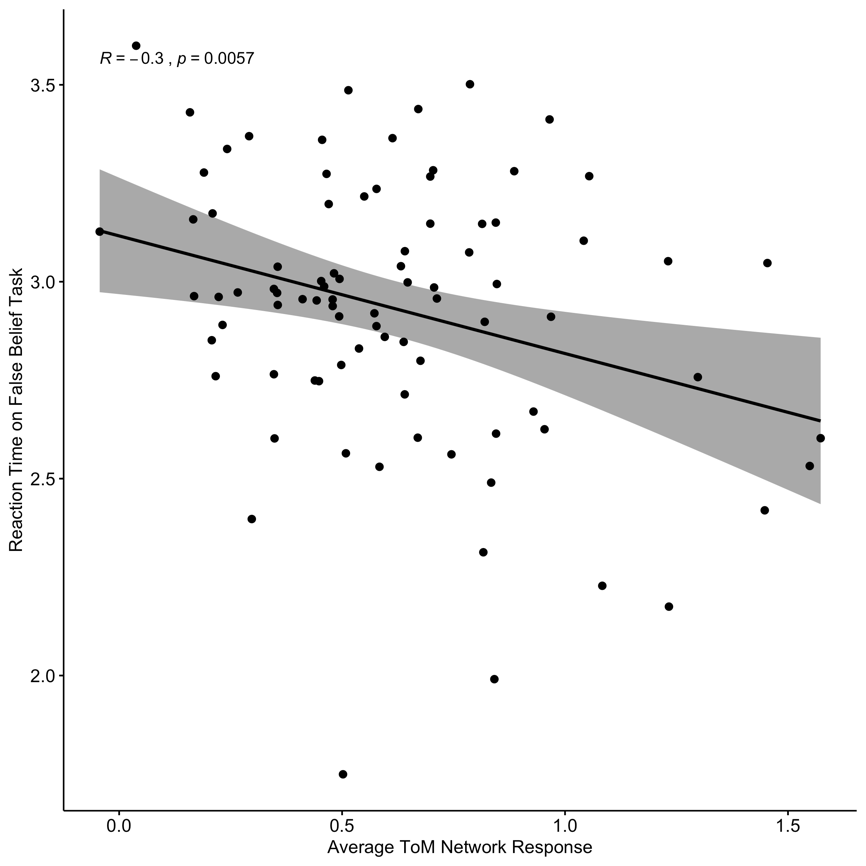


**Figure S12. Relationship between average Theory-of-Mind network response and accuracy and RT.** Accuracy is measured in proportion of correct responses and RT is in seconds. Brain responses are measured as the percent signal change in the ROI for the contrast Belief>Photo on the false belief task. The pattern of results for all individual ROIs was very similar for both accuracy and RT.

**
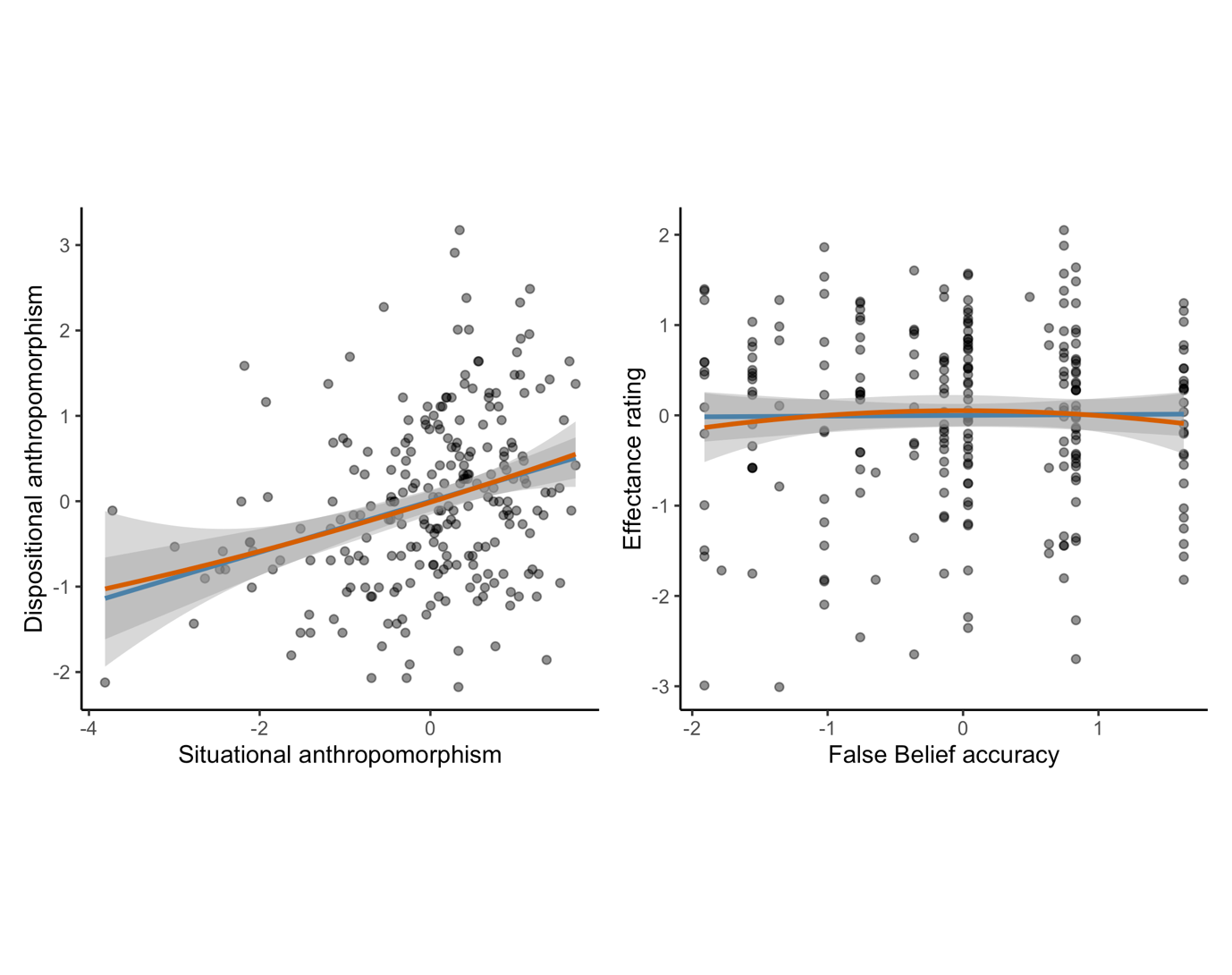
**

**Figure S13. Relationship between anthropomorphism and Theory-of-Mind indices.** While dispositional and situational anthropomorphism were positively correlated, no positive nor negative relation was found between effectance ratings and performance on the False Belief task. Indices are centered and scaled.


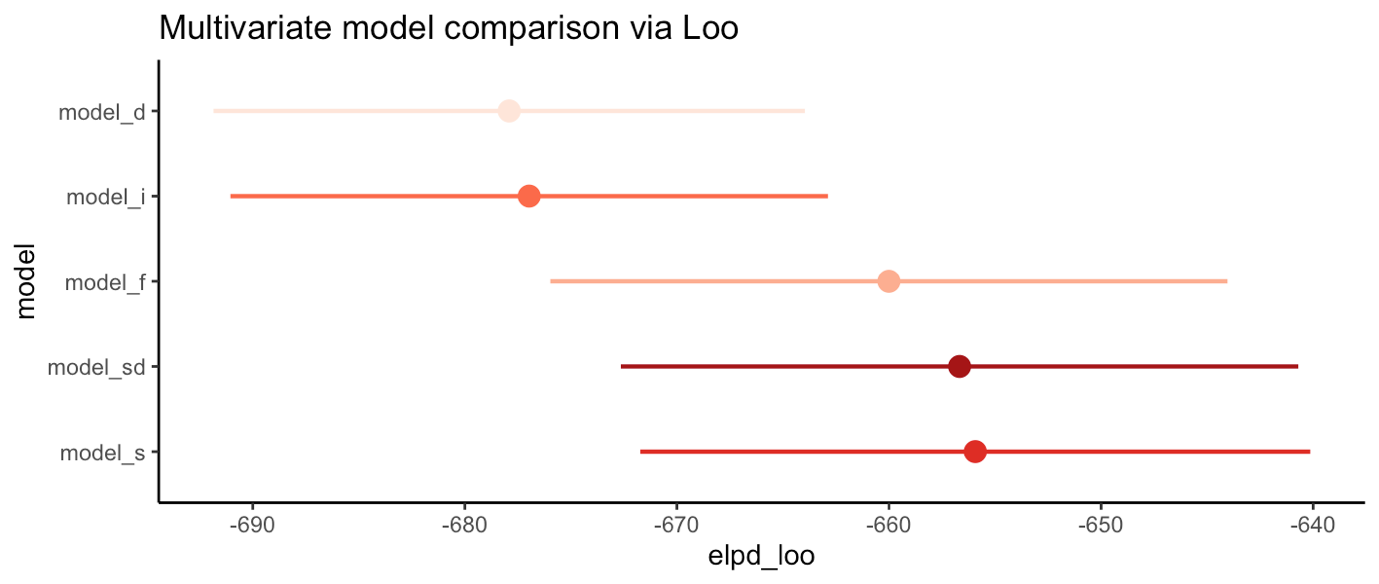


**Figure S14. Results from the leave-one-out cross-validation based on the posterior likelihood for estimated Bayesian multivariate regression models.** A model with only a linear situational anthropomorphism predictor provided the best fit (model_s; hypothesis 1), closely followed by a model with a linear situational and quadratic dispositional anthropomorphism predictor (model_sd), and a full model with linear and quadratic situational and dispositional anthropomorphism predictors (model_f). A model with a quadratic dispositional anthropomorphism predictor (model_d, hypothesis 2) overlapped with an intercept-only model (model_i). Dependent variables: effectance ratings and false belief performance.

| **Table S11. Results from the multivariate model comparison using leave-one-out cross-validation based on the posterior likelihood** | | | | | | | | |
| --- | --- | --- | --- | --- | --- | --- | --- | --- |
| *Models* | *elpd_diff* | *se_diff* | *elpd_loo* | *se_elpd_loo* | *p_loo* | *se_p_loo* | *looic* | *se_looic* |
| model_s | 0.0000 | 0.000 | -655.9 | 15.79 | 9.134 | 1.6020 | 1312 | 31.59 |
| model_sd | -0.7424 | 2.124 | -656.7 | 15.97 | 11.432 | 2.0880 | 1313 | 31.94 |
| model_f | -4.0750 | 2.305 | -660.0 | 15.96 | 15.368 | 2.3366 | 1320 | 31.93 |
| model_i | -21.0370 | 19.621 | -677.0 | 14.09 | 5.357 | 0.4405 | 1354 | 28.18 |
| model_d | -21.9786 | 9.633 | -677.9 | 13.94 | 6.989 | 0.5734 | 1356 | 27.88 |
| model_s: linear situational anthropomorphism predictor model, model_sd: linear situational and quadratic dispositional anthropomorphism predictor model, model_f: linear and quadratic situational and dispositional anthropomorphism predictors, model_i: intercept-only model, model_d: quadratic dispositional anthropomorphism predictor model. Dependent variables: effectance ratings and false belief performance. | | | | | | | | |


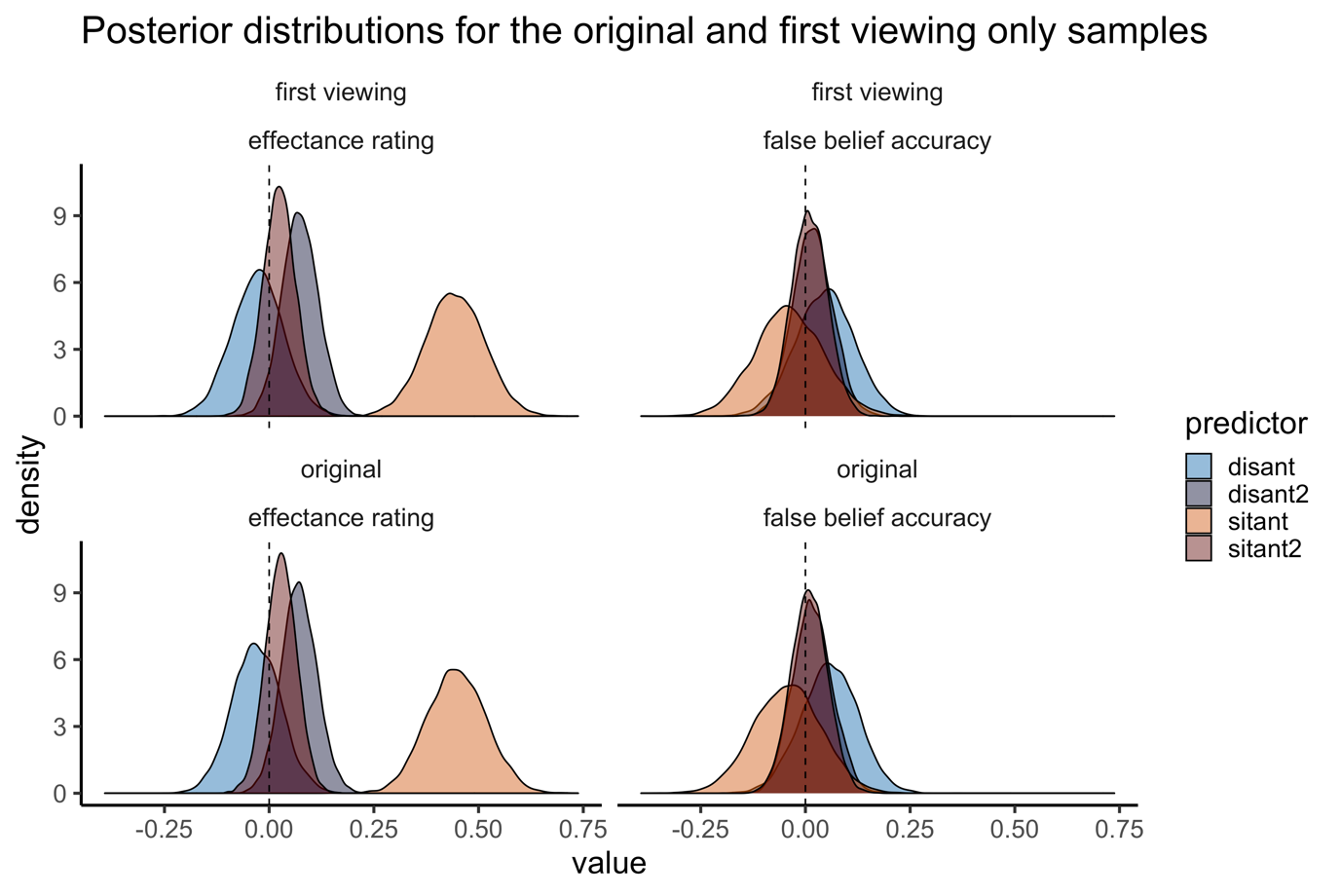
**Figure S15. Posterior distribution for the estimated Bayesian multivariate regression model with the original and first viewing sample only.** The full model was estimated with the original sample consisting of participants not meeting the preregistration exclusion criteria (original *n* = 241) and with an updated sample consisted of those participants that saw the film for the first time (first viewing sample *n* = 227). The posterior distributions obtained with these two samples overlap. Distant = linear dispositional anthropomorphism predictor, distant2 = quadratic dispositional anthropomorphism predictor, sitant = linear situational anthropomorphism predictor, sitant2 = quadratic situational anthropomorphism predictor.


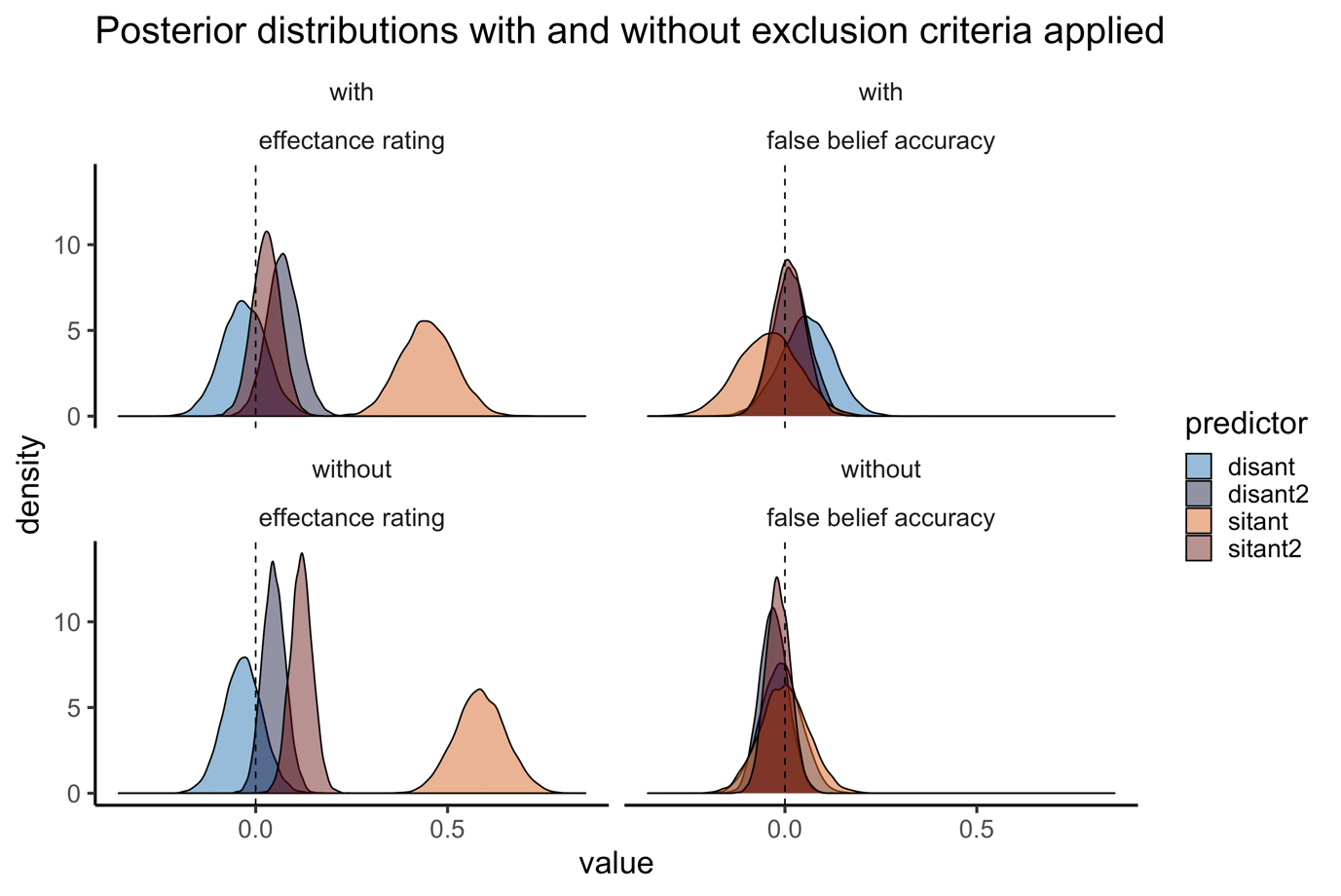


**Figure S16. Posterior distribution for the estimated Bayesian multivariate regression model with or without exclusion criteria applied.** When exclusion criteria were not applied (*n* = 311) similar results were obtained as when applied (*n* = 241). Distant = linear dispositional anthropomorphism predictor, distant2 = quadratic dispositional anthropomorphism predictor, sitant = linear situational anthropomorphism predictor, sitant2 = quadratic situational anthropomorphism predictor.

| **Table S12. Relationship between exploratory, situational and general Theory-of-Mind measures** | | |  |
| --- | --- | --- | --- |
| Effectance rating – Reliance on Theory-of-Mind | 0.18 [0.05 – 0.31] |  |  |
| Effectance rating – Accuracy of prediction | 0.12 [-0.01 – 0.25] |  |  |
| False belief performance – Reliance on Theory-of-Mind | 0.06 [-0.07 – 0.19] |  |  |
| False belief performance – Accuracy of prediction | 0.11 [-0.02 – 0.24] |  |  |
| Reliance on Theory-of-Mind – Accuracy of prediction | 0.40 [0.28 – 0.50] |  |  |
| Pearson’s r and 95% Confidence Interval reported. | | | |

**
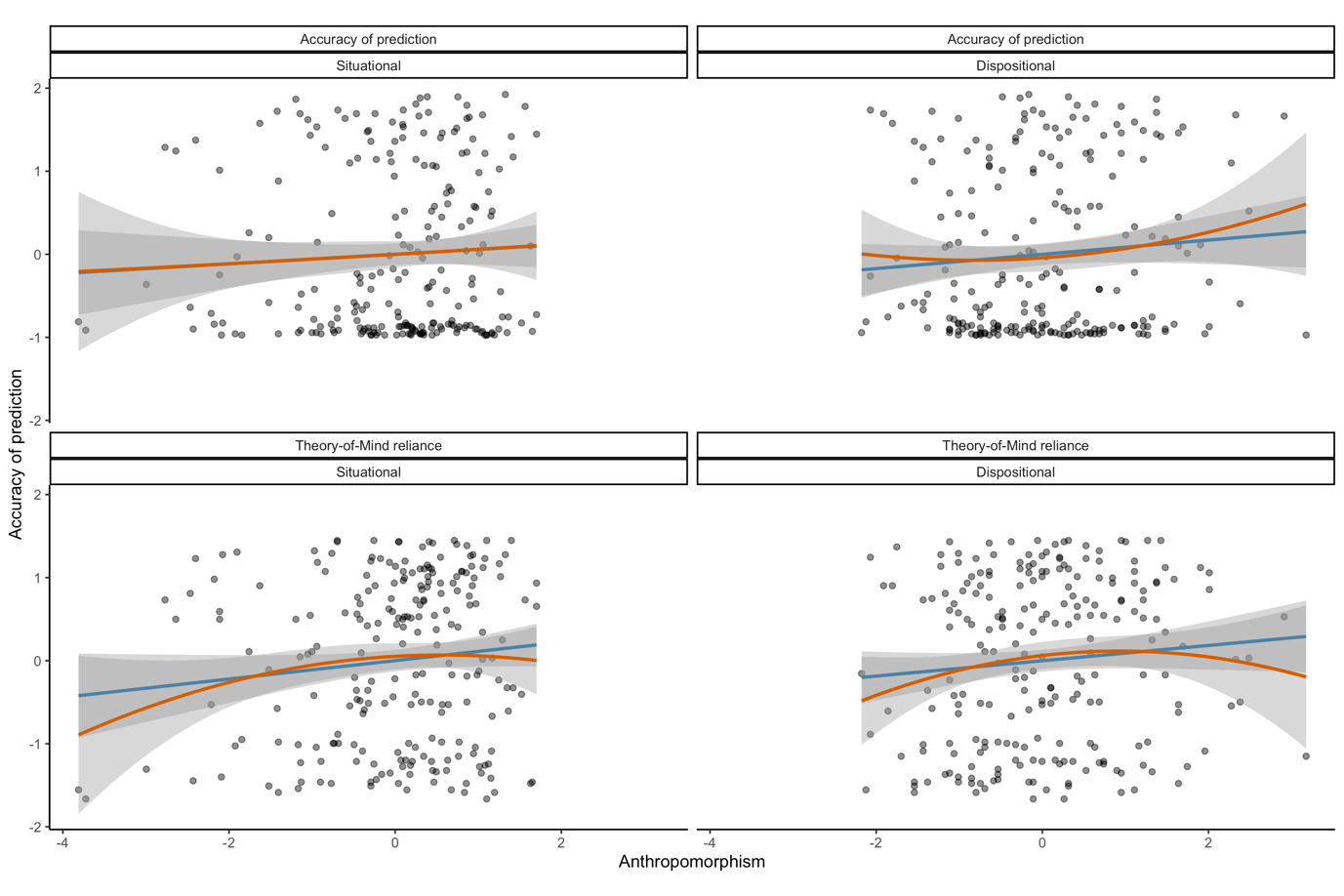
**

**Figure S17. Anthropomorphism and the open-ended prediction made during the film.** Neither situational nor dispositional anthropomorphism predicted the accuracy of the open-ended prediction made by the participant during the film or the reliance of this prediction on Theory-of-Mind (quadratic predictor in red, linear predictor in blue). Indices are centered and scaled.

| **Table S13. Estimated posterior regression coefficient for each predictor for the open-ended prediction made during the film** | | | | |
| --- | --- | --- | --- | --- |
|  | **Accuracy of the prediction** | | **Reliance on Theory-of-Mind** | |
| *Predictors* | *Estimates* | *CI (95%)* | *Estimates* | *CI (95%)* |
| Intercept | 0.07 | -0.04 – 0.18 | 0.10 | -0.07 – 0.26 |
| Linear situational | 0.01 | -0.06 – 0.07 | 0.02 | -0.08 – 0.12 |
| Quadratic situational | -0.01 | -0.05 – 0.02 | -0.02 | -0.09 – 0.04 |
| Linear dispositional | -0.00 | -0.05 – 0.06 | 0.02 | -0.07 – 0.11 |
| Quadratic dispositional | 0.00 | -0.04 – 0.03 | -0.03 | -0.09 – 0.04 |
| R^2^ Bayes | 0.002 | | 0.012 | |
| CI (95): 95% credibility interval. | | | | |

**References**

Allen, M., Poggiali, D., Whitaker, K., Marshall, T. R., & Kievit, R. A. (2019). Raincloud plots: A multi-platform tool for robust data visualization. *Wellcome Open Research*, *4*, 63. https://doi.org/10.12688/wellcomeopenres.15191.1

Cullen, H., Kanai, R., Bahrami, B., & Rees, G. (2014). Individual differences in anthropomorphic attributions and human brain structure. *Social Cognitive and Affective Neuroscience*, *9*(9), 1276–1280. https://doi.org/10.1093/scan/nst109

Darda, K. M., Butler, E. E., & Ramsey, R. (2018). Functional Specificity and Sex Differences in the Neural Circuits Supporting the Inhibition of Automatic Imitation. *Journal of Cognitive Neuroscience*, *30*(6), 914–933. https://doi.org/10.1162/jocn_a_01261

Dodell-Feder, D., Koster-Hale, J., Bedny, M., & Saxe, R. (2011). FMRI item analysis in a theory of mind task. *NeuroImage*, *55*(2), 705–712. https://doi.org/10.1016/j.neuroimage.2010.12.040

Downing, P. E., Wiggett, A. J., & Peelen, M. V. (2007). Functional Magnetic Resonance Imaging Investigation of Overlapping Lateral Occipitotemporal Activations Using Multi-Voxel Pattern Analysis. *Journal of Neuroscience*, *27*(1), 226–233. https://doi.org/10.1523/JNEUROSCI.3619-06.2007

Dufour, N., Redcay, E., Young, L., Mavros, P. L., Moran, J. M., Triantafyllou, C., Gabrieli, J. D. E., & Saxe, R. (2013). Similar Brain Activation during False Belief Tasks in a Large Sample of Adults with and without Autism. *PLoS ONE*, *8*(9). https://doi.org/10.1371/journal.pone.0075468

Eriksen, B. A., & Eriksen, C. W. (1974). Effects of noise letters upon the identification of a target letter in a nonsearch task. *Perception & Psychophysics*, *16*(1), 143–149. https://doi.org/10.3758/BF03203267

Esteban, O., Birman, D., Schaer, M., Koyejo, O. O., Poldrack, R. A., & Gorgolewski, K. J. (2017). MRIQC: Advancing the automatic prediction of image quality in MRI from unseen sites. *PLOS ONE*, *12*(9), e0184661. https://doi.org/10.1371/journal.pone.0184661

Jacoby, N., Bruneau, E., Koster-Hale, J., & Saxe, R. (2016). Localizing Pain Matrix and Theory of Mind networks with both verbal and non-verbal stimuli. *NeuroImage*, *126*, 39–48. https://doi.org/10.1016/j.neuroimage.2015.11.025

Kriegeskorte, N., Simmons, W. K., Bellgowan, P. S. F., & Baker, C. I. (2009). Circular analysis in systems neuroscience: The dangers of double dipping. *Nature Neuroscience*, *12*(5), 535–540. https://doi.org/10.1038/nn.2303

Lancaster, J. L., Tordesillas‐Gutiérrez, D., Martinez, M., Salinas, F., Evans, A., Zilles, K., Mazziotta, J. C., & Fox, P. T. (2007). Bias between MNI and Talairach coordinates analyzed using the ICBM-152 brain template. *Human Brain Mapping*, *28*(11), 1194–1205. https://doi.org/10.1002/hbm.20345

Nieto-Castañón, A., & Fedorenko, E. (2012). Subject-specific functional localizers increase sensitivity and functional resolution of multi-subject analyses. *NeuroImage*, *63*(3), 1646–1669. https://doi.org/10.1016/j.neuroimage.2012.06.065

Richardson, H., Lisandrelli, G., Riobueno-Naylor, A., & Saxe, R. (2018). Development of the social brain from age three to twelve years. *Nature Communications*, *9*(1), 1027. https://doi.org/10.1038/s41467-018-03399-2

Spunt, R. P., & Lieberman, M. D. (2012). Dissociating Modality-Specific and Supramodal Neural Systems for Action Understanding. *Journal of Neuroscience*, *32*(10), 3575–3583. https://doi.org/10.1523/JNEUROSCI.5715-11.2012

Waytz, A., Cacioppo, J., & Epley, N. (2010). Who Sees Human? The Stability and Importance of Individual Differences in Anthropomorphism. *Perspectives on Psychological Science*, *5*(3), 219–232. https://doi.org/10.1177/1745691610369336

Waytz, A., Morewedge, C. K., Epley, N., Monteleone, G., Gao, J.-H., & Cacioppo, J. T. (2010). Making sense by making sentient: Effectance motivation increases anthropomorphism. *Journal of Personality and Social Psychology*, *99*(3), 410–435. https://doi.org/10.1037/a0020240
